# No lysine? No problem: the surprising roles of WNK kinases in physiology and disease

**DOI:** 10.1042/BCJ20250110

**Published:** 2026-10-19

**Authors:** Ji-Ung Jung, Ankita B. Jaykumar, Clinton A. Taylor, Melanie H. Cobb

**Affiliations:** 1 Department of Pharmacology, UT Southwestern Medical Center, Dallas, TX 75390, U.S.A.; 2 Department of Pharmaceutical Sciences, University of North Texas Health, Fort Worth, TX 76107, U.S.A.

**Keywords:** brain, cancer, kinase, metabolic regulation, transport, WNK

## Abstract

WNK (With No lysine K) protein kinases gained early attention because gain-of-function mutations in genes encoding two of them cause hypertension. They differ from other kinases due to the unusual location of the catalytic lysine, hence the name WNK. These kinases control many aspects of ion transport in the kidney and are essential for development and maintenance of the cardiovascular system. In the present review, we describe WNK proteins and selected substrates, in particular, the kinases oxidative stress-responsive kinase 1 and SPS1-related proline/alanine-rich kinase, and their regulation. We spotlight findings that emphasize the broad relevance of WNKs in normal physiology and disease. Our overview focuses on basic aspects of ion transport that occur in many cell types and tissues, functions in the endothelial system, interactions with insulin-dependent and metabolic pathways, the importance in the brain in part through controlling chloride ion balance, and the whirlwind of findings in cancer that reveal how WNK signaling can be repurposed. Other actions that may be of equal importance, e.g., influences on the immune system, are touched on only briefly. We also note a few genome-wide association studies that have uncovered multiple SNPs in genes encoding WNKs and downstream effectors in an array of diseases, in anticipation of more to come in the future.

## Introduction

Twenty-five years ago, gain-of-function mutations in genes encoding the protein kinases WNK4 and WNK1 were uncovered as the cause of a rare genetic hypertension, pseudohypoaldosteronism type II (PHAII), also known as familial hyperkalemic hypertension (FHHt) [1]. Extensive efforts continue to elaborate on the actions of these kinases on ion balance in the kidney that can lead to hypertension and altered blood pressure. WNK1 is essential for the development and proper function of the cardiovascular system in mice [2]. Subsequently, loss-of-function mutations, in a WNK1 exon with restricted expression, were found to disrupt pain sensing in peripheral neurons [3]. Inappropriate WNK activity disturbs glucose handling and may augment metastasis in many cancers. Genome-wide association studies have also uncovered multiple SNPs in genes encoding WNKs and downstream effectors in diseases including neurocognitive deficits and impaired learning. Here, we present an overview of WNK (With No lysine K) proteins, their regulation, and recent physiological findings that account for some of their increasingly apparent roles in disease.

## WNK structure, regulation, and partners in ion homeostasis

### WNK structure and isoforms

The four WNK protein kinases, WNK1–WNK4, occupy their own branch of the human kinome tree along with their pseudokinase relatives, nuclear receptor-binding protein (NRBP) 1 and 2. The WNK acronym comes from the unique placement of the catalytic lysine within the active site. In almost all kinases, this lysine in strand β3 forms an ion pair with a conserved glutamate in helix αC, and this ‘K-E’ ion pair is required for catalysis. In WNKs, this lysine is instead found in strand β2 [4]. This unusual lysine position offers distinct regulatory potential and factors into the relatively high selectivity of several WNK inhibitors, which bind with low nanomolar affinity [5–7] (Table 1). In addition to the kinase domains, WNKs have few folded globular domains and coiled-coils interspersed among large, poorly conserved unstructured regions that act as scaffolds (Figure 1) [8,9]. WNK kinase domains lie near the N-termini immediately, followed by the first of two CCT-like (conserved C-terminal) domains (CCTL1) [10–14], which are related to CCT domains present in the downstream kinases, OSR1 (oxidative stress-responsive kinase 1) and SPAK (SPS1-related proline/alanine-rich kinase) (Figure 1). CCT domains bind WNKs, substrates, and a panoply of other proteins via consensus motifs R-F-x-V/I or R-x-F-x-V/I [10–12,15–20]. A second CCT-like domain (CCTL2) is found in the middle of all four WNKs [21,22]. Each WNK also has one or more OSR1/SPAK CCT binding motifs, with WNK1 having the most, with four [23,24]. Near the C-terminus, each WNK has an additional coiled-coil, also involved in oligomerization [8,9,24].

**Figure 1:**
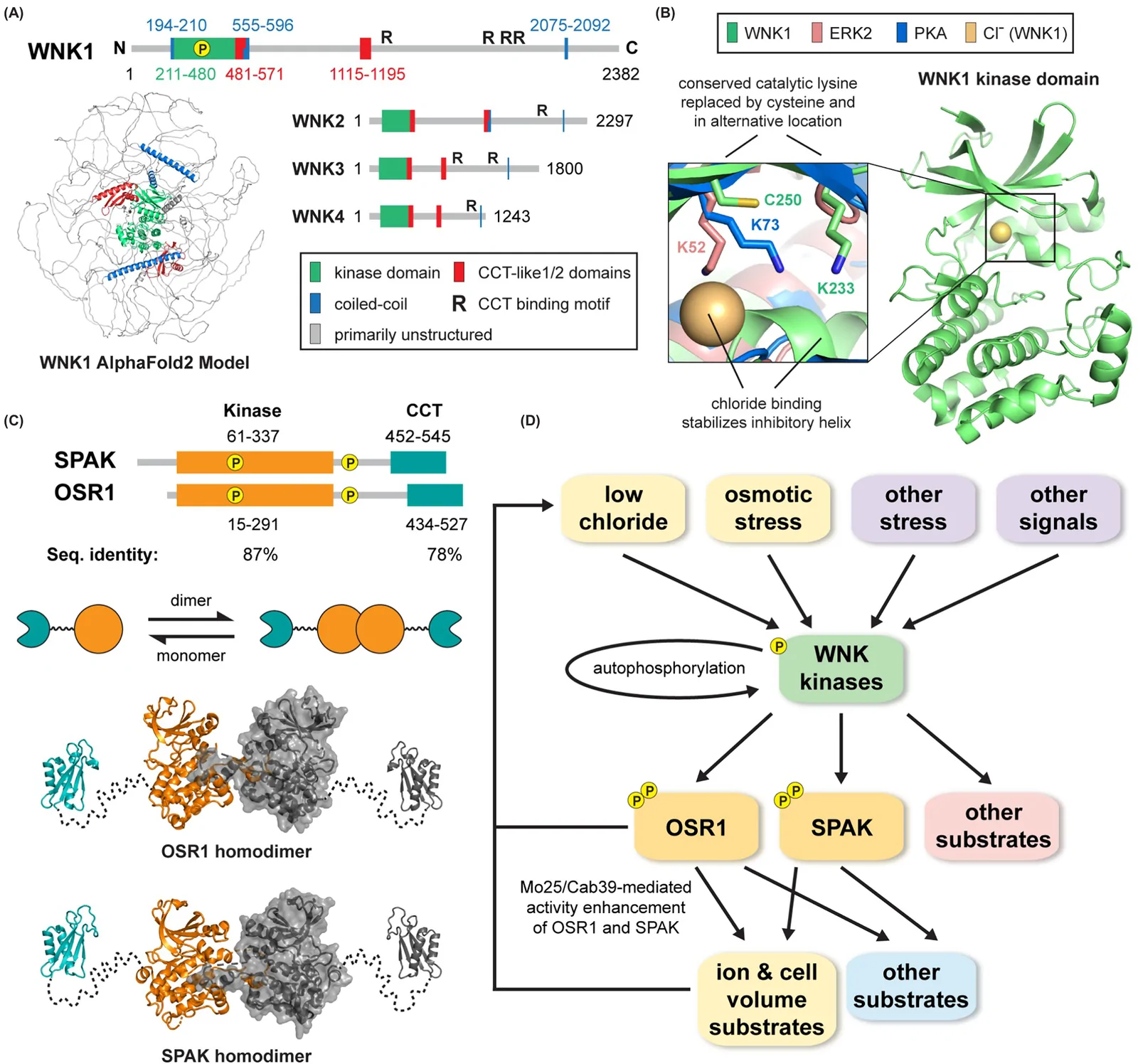
Overview of the WNK–OSR1/SPAK pathway (**A**) Domain organization of WNK kinases. (**B**) The crystal structure of the WNK1 kinase domain bound to chloride [31]. WNKs have a unique placement of the catalytic lysine, which facilitates chloride-mediated inhibition of kinase activity. The crystal structure of the active sites of ERK2 and cAMP-dependent protein kinase are shown for comparison (PDB: 4Q2A, 2ERK, and 1ATP). (**C**) Domain organization of the kinases OSR1 and SPAK, as well as the crystal structures of the dimeric activation loop domain-swapped kinase domains (PDB: 3DAK and 5D9H) [32–34]. (**D**) WNK–OSR1/SPAK pathway graphical summary of the core pathway components.

**Table 1:** WNK kinase inhibitors

Inhibitor	Target/mechanism	Inhibitory activity	Specificity/off-target effects	Ref.
WNK463	Pan-WNK ATP competitive inhibitor	IC_50_ (MBP): WNK1: 5 nM, WNK2: 1 nM WNK3: 6 nM WNK4: 9 nM IC_50_ (OSR1) WNK1: 41 nM IC_50_ (HEK293 cells pOSR1) WNK1: 106 nM	KINOMEScan reporter ligand binding assay: 2 of 442 kinases had >50% inhibition at 10 μM	[6]
WNK-IN-11	Allosteric non-ATP competitive inhibitor selective for WNK1 and WNK3	IC_50_ WNK1: 4 nM EC_50_ (HEK293 cells pOSR1) WNK1: 352 nM WNK1 selectivity vs… WNK2: 57-fold, WNK3: none, WNK4: ∼1000-fold	KINOMEScan reporter ligand binding assay: 5 of 440 kinases had >50% inhibition at 10 μM	[5]
WNK trihalo-sulfone compound 1	ATP competitive WNK1 selective	IC_50_ (GST-OSR1 314-34) WNK1: 1.6 μM IC_50_ (MDAMB231 pOSR1) WNK1: 4.3 μM WNK1 selectivity vs WNK3: 10-fold	Eurofins kinase specificity screen: ‘similar inhibition of 4 [of 46] kinases in diverse classes.’ 10 μM inhibitor	[25]
SW120619	ATP competitive WNK3 selective	IC_50_ (OSR1) WNK1: 2.3 μM IC_50_ (OSR1) WNK3: 0.7 μM	Eurofins kinase specificity screen: No inhibition of kinase activity >20% for 46 kinases tested. 10 μM inhibitor	[7]
SW182086	ATP competitive WNK1 and WNK3 selective	>50% inhibition of WNK1, WNK3, and WNK4 in Eurofins kinases specificity screen (10 μM inhibitor)	Eurofins kinase specificity screen: 2 kinases with inhibition of kinase activity >20% for 46 kinases tested. 10 μM inhibitor	[7]

Summary of properties of WNK kinase inhibitors [5–7,25,26]. Summary of OSR1/SPAK inhibitors [27–30] can be found in Supplemental Table S1.

WNKs have many common functions, many carried out by OSR1/SPAK, yet distinctions in tissue expression and isoforms empower their functional specialization appropriate to context. WNK1 is often referred to as L-WNK1 when the full-length transcript is meant, whereas KS-WNK1 denotes the kidney-specific shorter transcript that begins after exon 4a [35]. A striking example, WNK1, more than the other WNKs, has been found in nearly all cell types and tissues in mammals; yet WNK4 dominates ion control in the kidney in part because KS-WNK1, highly expressed in the distal tubule, initiates from an alternate start site and lacks most of the kinase domain, retaining only the scaffolding functions carried out by the C-terminal regions [36].

### WNK activation and turnover

Early work found that WNK1 immunoprecipitated from cells exposed to NaCl exhibited enhanced autophosphorylation, implying WNK1 is a salt-sensitive kinase [4]. Hyperosmotic stress and cell shrinkage, as well as hypo-osmotic stress, cell swelling, and low intracellular chloride activate WNKs to restore cell volume and maintain ion homeostasis (Figure 2) [4,37–40]. WNK activation is paralleled by autophosphorylation on the activation loop serine (Ser382 in WNK1) [14]. High osmolarity (osmotic pressure) shifts the WNK1 and WNK3 kinase domains from inhibited chloride-binding dimers to activatable monomers [41]. This is presumed to be a property of many WNKs. Cellular crowding-induced WNK phase separation, an effect of hyperosmotic stress, activates WNKs [8]. The Goldsmith lab showed that chloride inhibition occurs through binding to an inhibitory helix in the kinase domain activation loop, providing a physical basis for the unique placement of the catalytic lysine, which otherwise would sterically clash with the chloride binding site (Figure 1) [31,^42^]. Like chloride, water itself can function as an allosteric ligand in WNK kinase domains, as a conserved water network organized by charged residues likely functions in osmosensing [41,43,44]. WNK1 is also activated by hypo-osmotic conditions by a mechanism requiring increased intracellular calcium and the Piezo1 mechanosensitive ion channel [45].

**Figure 2:**
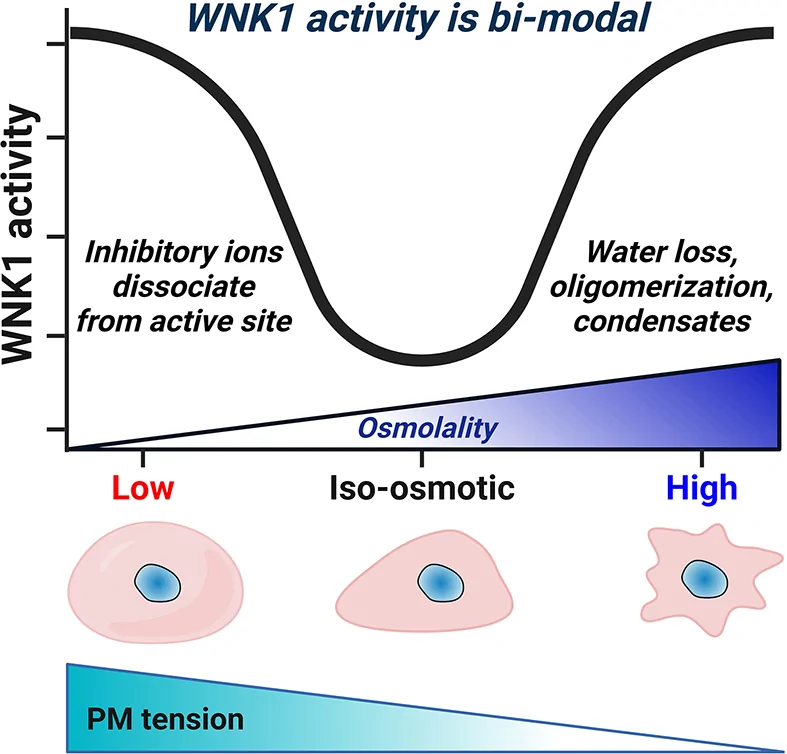
Regulation of WNK1 activation and cell volume changes by osmolality Hypotonic conditions, which result in low osmolality and cell swelling with increased membrane tension, cause inhibitory ions, particularly Cl^−^, to dissociate from the WNK1 active site, thereby enhancing its kinase activity. Increased intracellular calcium and the mechanosensitive channel Piezo1 are required for the hypotonic response [45]. High osmolality from hypertonic conditions and water loss that causes cell shrinkage and reduced membrane tension, triggers WNK1 oligomerization, thereby enhancing its activation.

Disrupting regulated turnover of WNK proteins can lead to PHAII [46]. All four WNKs can be degraded by Cullin3 (CUL3) Ring ubiquitin ligase (CRL3) complexes, which utilize CUL3 and its substrate-trapping adaptors, primarily KLHL3 (kelch-like protein 3) and also KLHL2 [47–50]. Human mutations in WNK4 that interfere with its recognition by KLHL3 result in increased WNK4 protein amounts in the kidney, particularly within the distal convoluted tubule, causing PHAII [1]. Mutations have also been identified that affect turnover of the kidney-specific short form of WNK1, KS-WNK1 [51]. Mutations in CUL3 and KLHL3 themselves can also cause hypertension due to reduced WNK degradation [52]. Furthermore, modifications of the CRL3 components have been identified that promote WNK accumulation. These include phosphorylation of KLHL3 by protein kinase C, cAMP-dependent protein kinase, and AKT, providing regulatory inputs that can enhance WNK function from an array of hormonal signals including vasopressin and insulin [53–55]. O-GlcNAcylation of KLHL proteins and WNKs, identified in HEK293T cell-based overexpression systems, has also been shown to affect WNK accumulation [56].

In addition to the CRL3–KLHL3 pathway, WNK kinases are also turned over by other E3 ubiquitin ligases, including NEDD4-2, SMURFs, and UBR5 [57–59]. NEDD4-2 ubiquitinates WNK kinases, leading to their degradation; the activity of NEDD4-2 is tightly regulated by stress and hormonal signals like aldosterone through kinases like SGK1 (serum/glucocorticoid-regulated kinase 1). In the kidney, WNK1 activates SGK1, which phosphorylates NEDD4-2 to inhibit its ubiquitin ligase function. This pathway has been supported by cell-based studies, including HEK293 and WNK1-deficient systems, as well as mouse models [60–62]. Another NEDD4-2 substrate is the epithelial Na^+^ channel (ENaC), the target of amiloride diuretics. The dual action of NEDD4-2 creates a feedback loop where WNK1 activates SGK1, which in turn suppresses NEDD4-2, allowing increased WNK and stabilizing ENaC, increasing sodium reabsorption [63,64]. SGK1 can phosphorylate WNK1 on Thr60, although the function is unknown [65]. WNK1 association with mTORC2 has been suggested to contribute to activation of SGK1 and ENaC [66]. UBR5, on the other hand, has no reported connection to ion transport and orchestrates WNK turnover mainly by directing them to the lysosome, a non-proteasomal mechanism for controlling WNK stability [59]. Together, these pathways provide precise and dynamic control of WNK kinases in response to cellular and environmental cues (Figure 3).

**Figure 3:**
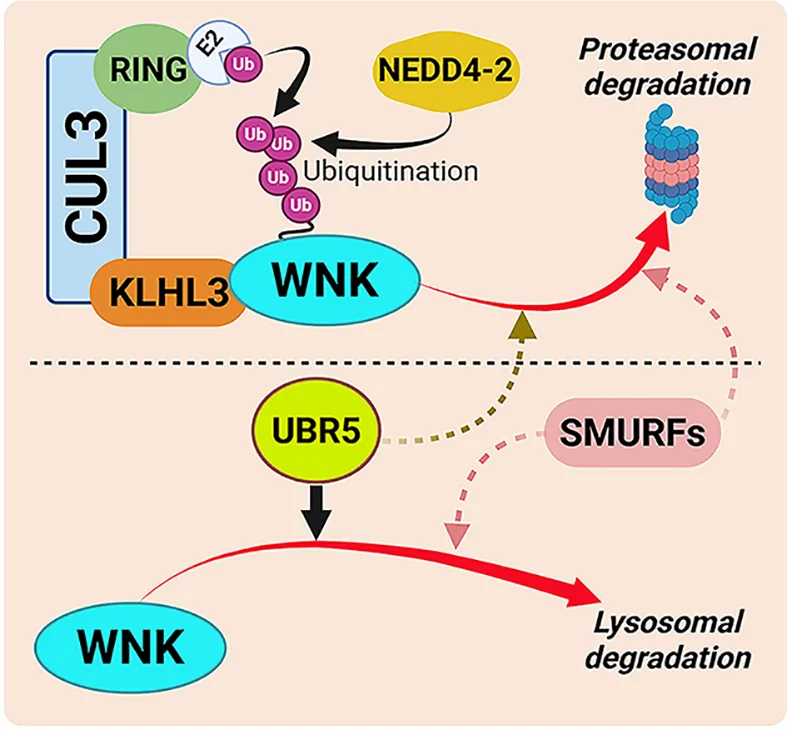
WNK kinase degradation pathways WNK kinases undergo ubiquitination and degradation through multiple regulatory routes involving distinct E3 ubiquitin ligase complexes. The CUL3–KLHL3 complex mediates WNK ubiquitination, leading to proteasomal degradation, whereas UBR5 promotes WNK turnover via lysosomal degradation. Additionally, NEDD4-2 and SMURF E3 ligases interact with WNK1 in the transforming growth factor-β (TGF-β) pathway and contribute to both proteasomal and lysosomal degradation pathways. Predictably, WNK1 displays nonlinear decay kinetics, indicating multiple temporally distinct degradative processes [59].

### OSR1/SPAK as downstream effectors

OSR1 and SPAK are major downstream effector kinases directly activated by WNK phosphorylation (Figure 1). WNKs scaffold many proteins and have other substrates, but defined phosphoregulation is often attributable to OSR1 and SPAK. OSR1 and SPAK are paralogs, with SPAK the product of a gene duplication during vertebrate evolution [67]. They possess an N-terminal kinase domain followed by a linker region with multiple regulatory sites and a CCT domain that binds R-F-x-V/I or R-x-F-x-V/I motifs [13,16–19]. The kinases have two primary sites of phosphorylation by WNKs [23,68,69], one within the activation loop and a second in the linker region. The linker region also binds the scaffold Mo25/Cab39 (calcium-binding protein 39), which can enhance kinase activity [70–73]. Phosphorylation at the linker site is the most common readout for WNK activity. Occasional differences in phosphorylation of the two kinases in response to WNK-activating stimuli have been noted, although there are few explanations of how or why these differences arise [20,45,74,75]. Certain other kinases, including mTORC2 and the DNA damage kinase ATM, can also phosphorylate OSR1 or SPAK and may act cooperatively or independently of WNK1 [76,77]. On the other hand, the mitogen-activated protein kinase p38, an ancient responder to osmotic stress, has been linked to the pathway but does not appear to be central to WNK1/OSR1/SPAK signaling [78].

As mediators of WNK action, OSR1 and SPAK regulate ion balance by phosphorylating the SLC12 family of cation-chloride co-transporters [4,23,39,31,79,80]. The sodium chloride cotransporter NCC (Na^+^ Cl^-^ cotransporter), key to dysregulation in PHAII, and the sodium potassium chloride transporter NKCC2 are activated by OSR1/SPAK and essential for maintaining ion balance in the kidney. WNK/OSR1/SPAK also activate NKCC1, which is widely expressed, important in cell volume maintenance and cell migration, and essential for hearing [81,82]. In contrast, OSR1/SPAK inhibit potassium-chloride co-transporters, thereby suppressing KCC (K^+^ Cl^−^ cotransporter) activity and reducing K^+^ and Cl^−^ efflux [83,84]. This inhibitory mechanism counterbalances NKCC (Na^+^ K^+^ 2Cl^-^ cotransporter) activation, enabling fine-tuned regulation of cellular and systemic ion and volume homeostasis [85,86]. Extensive reviews delineate the multifaceted actions of WNK1 and WNK4 in hypertension due to dysregulation of renal ion balance [46,87,88]. Disturbances in WNK-regulated K^+^ and Cl^−^ homeostasis are involved in many other systemic dysfunctions, including those in the nervous system (see below) and the immune system [89].

In addition to WNK functions in renal ion homeostasis, in many cells WNKs are organizers of regulatory volume increase (RVI), a rapid adaptive response that enables recovery from hypertonic stress-induced shrinkage. WNK1 rapidly undergoes liquid–liquid phase separation under hypertonic stress, forming condensates that activate OSR1/SPAK and enhance their phosphorylation of NKCC1 and KCCs to restore cell volume [8]. In contexts such as glioma, WNK3 predominates in NKCC1-dependent RVI and migration [90]. WNK1 activity is further modulated by conserved partners [20,74,91–93], including TSC22D (TSC22 domain family protein) proteins, transcription factors originally linked to TGF-β, and NRBP1, which can also act as a chaperone component of E3 ligases [94]. Through direct interactions with WNK1, these proteins stabilize the WNK1 complex, may facilitate condensate formation, may alter WNK1 structure, and promote WNK1 autophosphorylation at Ser382, thereby increasing its activity and fine-tuning RVI to maintain ion homeostasis under hypertonic conditions [74,91]. SPAK can also promote phase separation of WNK1 and TSC22D proteins (Taylor et al., unpublished). Interactions of SPAK CCT domains with binding motifs in the proteins accelerate phase separation. The WNK-related machinery involved in cell volume regulation is evolutionarily conserved across metazoans, highlighting the fundamental importance of this process for cell survival [74].

## WNKs in endothelial and vascular function

### WNKs in endothelial cells

Endothelial cells, which line the interior of blood vessels, are vital for vascular development, regulating blood pressure, and maintaining barrier integrity. Following up on the original finding that disruption of the WNK1 gene in mice resulted in embryonic death due to cardiovascular defects [95], Xie *et al.* in 2009 examined the conditional deletion of WNK1 from the endothelium [2]. Mice did not survive beyond E12 and displayed smaller heart chambers, impaired myocardial trabeculation, and defective angiogenesis in the yolk sac and vessels, confirming that cardiovascular development depends on endothelial WNK1.

Among the four WNK family members, WNK1 is the best-characterized isoform in the cardiovascular system and is the major WNK mRNA expressed in endothelial cells, including human umbilical vein endothelial cells (HUVECs) and human dermal microvascular endothelial cells (HDMECs), where it regulates angiogenesis, vascular tone, and mechanosensitive signaling [45,80,96]. WNK3 has also been reported in endothelial cells, although at much lower levels than WNK1, and its cardiovascular functions remain less well defined [80]. WNK4, in contrast, has not been detected in endothelial cells but has an established role in vascular smooth muscle, where it restricts TRPC3-mediated Ca^2+^ influx to regulate vascular tone and blood pressure [97,98]. WNK2 is predominantly expressed in the heart, brain, and colon at the tissue level [99], but its mRNA is undetectable in cultured endothelial cells [80], and its functional role in cardiovascular biology remains unclear. As other WNKs are expressed in the endothelium, these findings imply that WNK1 has functions that are not common with or cannot be replaced by the other family members [80], but this still leaves open the question of whether greater expression of WNK2–4 might compensate for WNK1 deletion. Consistent with this non-redundancy, the lethality of the WNK1 deletion was rescued by endothelial-specific expression of constitutively active OSR1 [2,96], indicating the large load carried by OSR1/SPAK in mediating WNK1 action and further implying specific connections between WNK1 and its downstream effectors that are not fully encompassed by the other WNKs.

The Cl^−^ channel ClC-5 has been implicated in regulating WNK1-dependent signaling by altering intracellular Cl^−^ concentration under low-chloride conditions, thereby modulating WNK1–RhoA activity and suppressing the endothelial AKT/eNOS pathway, ultimately influencing vascular tone and endothelial function [100]. These findings highlight a mechanistic link between Cl^−^ channel activity, WNK1 signaling, and endothelium-dependent vasodilation. Consistently, a recent study demonstrated that WNK1 also modulates arterial contractility through a chloride-dependent mechanism. Upon acetylcholine stimulation, activation of the TMEM16A Cl^−^ channel in endothelial cells reduces intracellular Cl^−^ levels, leading to activation of WNK1 and OSR1. This signaling cascade enhances the activity of the calcium channel TRPV4, amplifying Ca^2+^ signaling and promoting endothelium-dependent vasodilation [101].

Cells in the vascular system are continuously exposed to mechanical forces that originate from blood flow and muscle contractions, as well as external influences like osmotic stress. Osmotic stress arises from an imbalance in solute concentrations between the intracellular and extracellular environments, leading to water movement across the membrane and changes in membrane tension [102,103]. To restore balance and maintain homeostasis, cells respond by regulating ion transport, accumulating osmolytes, modulating water channel activity, and altering gene expression, all of which are influenced by WNKs. As noted above, WNKs detect changes in osmotic pressure and trigger cellular responses through OSR1/SPAK, ion cotransporters, and other molecules as appropriate, to adjust ion fluxes and restore osmotic balance (Figure 1) [39,104].

Although NCC is not expressed in the vascular system, NKCC1 and KCC pathways, regulated by WNK–OSR1/SPAK signaling, contribute to endothelial and vascular adaptation to osmotic and mechanical stress [105–109]. Recent work has further identified WNKs as key regulators of vacuolation under osmotic stress. In hypotonic environments, vacuole formation appears to result from decreased intracellular Cl^−^ during recovery from cell swelling, a process that imposes mechanical stress on the cell membrane. This vacuolation is suppressed in WNK1/WNK3 double knockout cells or upon treatment with the pan-WNK inhibitor WNK463 [110] (Table 1). Whether a similar WNK-dependent vacuolation response occurs in vascular endothelial or smooth muscle cells has not been tested, but these findings illustrate a general mechanism by which WNKs couple osmotic sensing to membrane mechanics that could plausibly extend to vascular cell types.

Shear stress results from the frictional force generated by blood flow. Changes in shear stress alter signaling, gene expression, cell shape, and vascular remodeling, including cell sprouting, proliferation, and migration [111–114]. Shear stress influences the progression of atherosclerotic plaques, particularly in regions of disturbed blood flow characterized by low shear stress, which may lead to increased vascular inflammation and permeability, thereby promoting atherosclerosis [115–117]. WNKs respond to shear stress to maintain endothelial homeostasis under mechanical stress ([118]; Jung et al., unpublished). Pharmacological inhibition of the SPAK–NKCC1 complex attenuates reactive astrogliosis and blood–brain barrier disruption and restores cerebral blood flow in a mouse model of vascular cognitive impairment caused by chronic hypoperfusion, highlighting a WNK–SPAK signaling axis linked to vascular dysfunction [119]. A recent study revealed that WNK1 is enriched in regions of low shear stress in the mouse endothelium compared with areas of high shear stress, and in endothelium associated with human atherosclerotic plaques. In zebrafish, *wnk1a* negatively regulated endothelial cell proliferation under no-flow conditions, highlighting a potential role for WNK1 signaling in vascular homeostasis in response to flow-derived mechanical stress [118]. This appears to contrast with earlier findings that WNK1 knockdown reduces proliferation and migration in cultured human endothelial cells [80], a difference that may reflect at least two factors. Zebrafish possess two non-redundant *wnk1* paralogs (*wnk1a* and *wnk1b*) [120], unlike the single WNK1 gene in humans, and endothelial shear responses are magnitude- and dynamics-dependent, such that static culture and acute *in vivo* flow cessation represent distinct mechanical contexts [121,122]. WNK1's role in endothelial proliferation may therefore be context-dependent rather than uniformly pro- or anti-proliferative.

In response to shear stress, the mechanosensitive ion channel Piezo1 senses flow-driven mechanical forces, becomes activated, and converts these mechanical forces into intracellular signaling [123]. A proteomic analysis of Hereditary Stomatocytosis patients with Piezo1 gain-of-function mutations identified increased abundance of WNK1 and OSR1. Additionally, OSR1 phosphoThr185, a site phosphorylated by WNK1, was elevated in patients carrying Piezo1 mutations [124]. Recent evidence suggests that Piezo1 regulates WNK1 indirectly through Ca^2+^ influx rather than by direct interaction [45]. In endothelial cells, Piezo1 activation by Yoda1 or hypotonic stress induces Ca^2+^-dependent phosphorylation of WNK1 and its downstream targets OSR1/SPAK, and this response is lost when intracellular Ca^2+^ is chelated or Piezo1 is depleted. These findings support a Piezo1–Ca^2+^–WNK1 axis in vascular cells, although whether other Ca^2+^ channels can engage WNK1 in a similar manner remains to be determined. Piezo1-mediated Ca^2+^ influx is critical for downstream effectors, as observed in non-vascular cells where hypotonic swelling activates Piezo1 to trigger Ca^2+^-dependent activation of K^2+^ channels (IK/BK) for regulatory volume decrease (RVD) [125], reminiscent of WNKs’ role in sensing cell volume changes and maintaining ion homeostasis [83]. These findings collectively emphasize the importance of mechanosensitive WNK1 signaling in vascular function and homeostasis and, with further study, will expand understanding of molecular mechanisms that regulate endothelial responses to mechanical stress.

Transient receptor potential cation channel subfamily V (TRPV) members are calcium (Ca^2+^)-permeable ion channels that respond to mechanical stimuli and are involved in mechanosensation [126,127]. TRPV4 is essential for blood vessel dilation and the formation of new blood vessels in response to fluid flow [128]. TRPV4 is also involved in pathological conditions that lead to endothelial dysfunction and disruption of cellular structure [129,130]. Shear stress-mediated Ca^2+^ influx triggers the activation of TRPV4 [131]. Notably, WNK1 regulates both the function and surface trafficking of TRPV4, contributing to osmosensing and the osmotically responsive signal transduction cascade in the distal nephron [132,133]. In endothelial cells, WNK1 modulates TRPV4 in a context-dependent manner. Under physiological conditions, WNK1–OSR1/SPAK signaling enhances TRPV4-mediated Ca^2+^ entry and supports vasodilation [101]. In contrast, during pathological inflammation such as pneumonia, loss of WNK1 activity can permit excessive TRPV4-mediated Ca^2+^ influx, contributing to endothelial barrier disruption [134]. WNK modulation of TRPV4 also impacts synaptic processes [132] (see below). Another TRP family member, TRPV1, is also expressed in vascular endothelial cells, aiding control of blood pressure and vascular function. Shear stress has been shown to modulate TRPV1 expression in HUVECs [135]. TRPV1 contributes to the modulation of the nitric oxide (NO) pathway and other signaling mechanisms in response to shear stress, thereby influencing vascular homeostasis [136]. Evidence from non-vascular tissues shows that TRPV1 activation triggers a Ca^2+^-dependent cascade involving PI3K (phosphoinositide 3-kinase), ERK, AKT, and WNK, enhancing NKCC1 activity to drive RVI, as seen in the lens epithelium [137]. Additional studies in other epithelial systems indicate that WNKs modulate TRPV5 and TRPC6 [138–140], suggesting that WNK-dependent regulation of TRP channels is a conserved mechanism fine-tuning ion transport.

In vascular systems, Piezo1 and TRP channels, particularly TRPV4, form a coordinated mechanosensory network that translates mechanical forces into Ca^2+^-dependent signaling and ATP release, supporting mechanosensation in diverse tissues, including the specialized epithelium lining the urinary tract and chondrocytes [141,142]. Piezo1 functions as a primary sensor of membrane stretch or shear stress, while TRPV4 amplifies and sustains downstream calcium responses, contributing to vasorelaxation and context-dependent regulation of endothelial barrier function [131,143,144]. In pathophysiological contexts such as pressure-induced pancreatitis, pulmonary hypertension, or preeclampsia, dysregulated Piezo1–TRPV4 signaling leads to sustained Ca^2+^ elevation, cytoskeletal remodeling, mitochondrial dysfunction, and endothelial impairment [145–147]. This process operates alongside modulatory channels, including BK channels, which colocalize with Piezo1 to facilitate Ca^2+^-dependent vascular responses, and other mechanosensors such as TRPV2 and TRPM4 [146,148]. Together, these studies highlight the Piezo1–TRP network as a central mechanism for vascular mechanotransduction, barrier maintenance, and adaptive responses under physiological and pathological mechanical or osmotic stress. Whether WNK kinases are functionally integrated into this network in vascular cells, beyond the Piezo1–WNK1 and TRPV4–WNK1 links described above, remains to be established.

WNK1 may also contribute to endothelial mechanosensitivity by modulating inward-rectifier potassium channels, particularly Kir2.1, although this link has so far only been demonstrated in a heterologous HEK293 expression system rather than in native vascular cells [19]. In this system, WNK1 phosphorylates OSR1, which directly recognizes the conserved R-x-F-x-V/I binding motif in Kir2.1, leading to enhanced membrane localization and channel activity. In parallel, endothelial Kir2.1 channels act as ‘end-stage boosters,’ amplifying calcium-dependent intermediate- and small-conductance potassium (IK and SK) channel-mediated vasodilation [149] and responding rapidly to mechanical stimuli such as shear stress and osmotic stress-induced membrane stretch [150,151]. Whether WNK1–OSR1 signaling regulates Kir2.1 in native endothelial cells to influence these mechanosensitive responses remains to be directly tested.

Angiogenesis, blood vessel expansion, and repair depend on the reorganization of endothelial junctions. Inhibition of WNK1 kinase activity with small-molecule inhibitors blocks endothelial sprouting and vessel extension in *ex vivo* mouse embryos [2,152]. TGF-β oversees both vessel integrity and angiogenesis. WNK1 cooperates with TGF-β signaling components in these functions. WNK1 protects turnover of TGF-β receptors and their SMAD transcription factors and stabilizes the tyrosine kinase receptor AXL, involved in both cell migration and angiogenesis [152]. In contrast, WNK1 inhibition promotes TGF-β-dependent degradation of AXL. OSR1 interacts with the tight junction protein occludin in promoting tight junction turnover, an additional mechanism contributing to TGF-β-regulated angiogenesis. In zebrafish, WNK1 isoforms (*wnk1a* and *wnk1b*) are observed in the posterior cardinal vein. WNK1 knockdown leads to angiogenesis defects similar to those seen with vascular endothelial growth factor receptor 2 (VEGFR2) knockdown. Furthermore, a reduction in VEGFR2 or VEGFR3 decreases *wnk1a* expression, linking VEGF/VEGFR signaling to both the activation and transcriptional regulation of WNK1 during angiogenesis [120].

Beyond the vascular wall, WNK signaling is also regulated systemically through Piezo1-dependent modulation of the ubiquitin ligase KLHL3. In red blood cells and the kidney, Piezo1 activation enhances KLHL3-mediated WNK ubiquitination and degradation, thereby modulating cotransporters and channels, red blood cell volume, and renal K^+^ excretion [153]. Disruption of this pathway impairs WNK-mediated K^+^ handling and causes hyperkalemia, highlighting WNK coordination of systemic ionic homeostasis. Together, these findings illustrate how WNK–OSR1/SPAK signaling coordinates endothelial mechanotransduction, vascular development, and vascular tone, while also linking endothelial and smooth muscle functions in vascular remodeling (Figure 4).

**Figure 4:**
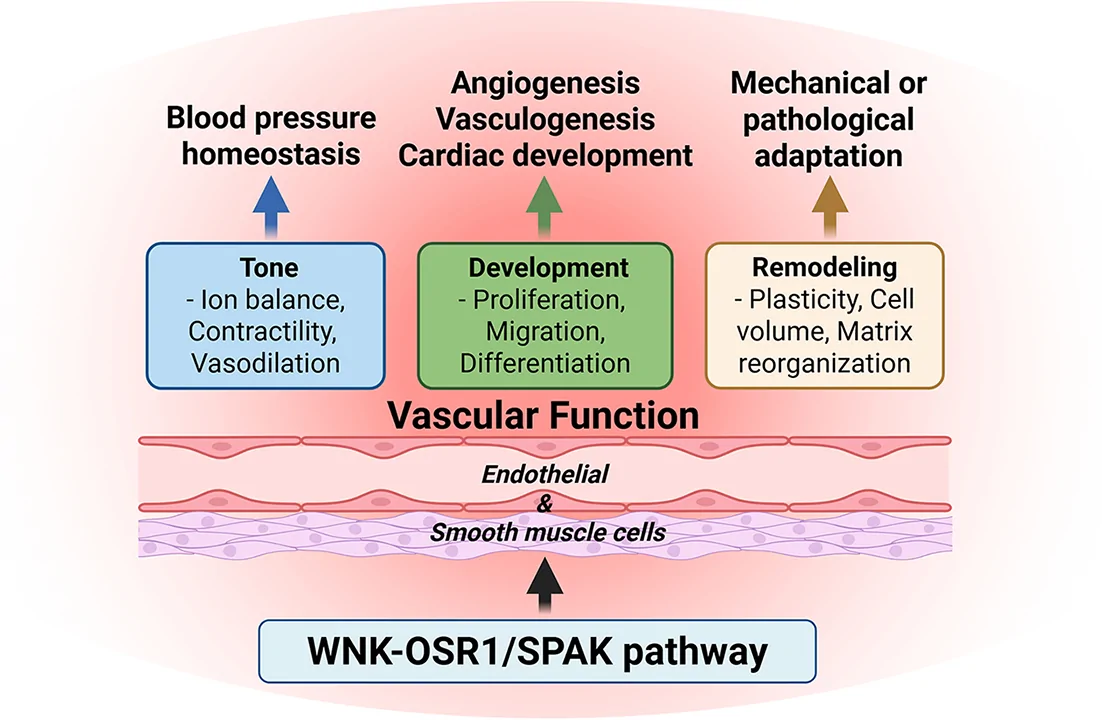
The WNK–OSR1/SPAK pathway is critical for maintaining vascular function WNKs support vascular function through both endothelial and smooth muscle cells via three major processes: regulation of vascular tone, promotion of vascular development, and facilitation of vascular remodeling. They govern ion balance, contractility, and vasodilation/vasoconstriction to maintain vascular tone; promotes endothelial and vascular cell growth, migration, and differentiation through growth factor-driven and mesenchymal transition-related pathways to support angiogenesis, vasculogenesis, and cardiac development; and modulates cell volume, plasticity, and matrix reorganization in both cell layers to enable mechanical and pathological vascular adaptation.

### WNKs in vascular smooth muscle cells

In contrast to endothelial cells, where WNK kinases have been implicated in mechanotransduction and endothelium-dependent vascular responses, WNK signaling in vascular smooth muscle cells (VSMCs) directly regulates vascular tone, contractility, and structural remodeling. This distinction underscores the cell type–specific compartmentalization of WNK function within the vascular wall.

VSMCs exhibit a distinct WNK isoform profile compared with endothelial cells. WNK4, which is expressed in VSMCs but is not readily detected in endothelial cells, restricts TRPC3-mediated Ca^2+^ influx and thereby limits α1-adrenergic agonist-induced vasoconstriction in small mesenteric arteries [97]. WNK1 also has a direct physiological role in VSMC contractility. WNK1 contributes to α1-adrenergic receptor–induced vasoconstriction, and heterozygous *Wnk1* deletion reduces arterial SPAK/NKCC1 phosphorylation and blood pressure responses [154]. Heterozygous *Wnk1* deletion also impairs pressure-induced myogenic tone in mesenteric arteries, an intrinsic smooth muscle contractile response independent of receptor stimulation, further supporting a direct role for WNK1 in VSMC contractility rather than solely endothelium-dependent mechanisms [155]. Consistently, VSMC-specific *Wnk1* deletion promotes a pathological switch away from the contractile phenotype and induces aortitis, highlighting its role in maintaining contractile identity [156]. WNK3 regulates vascular smooth muscle function through the WNK3–SPAK–NKCC1 cascade. Global WNK3 deletion prevents angiotensin II- or low-salt diet–induced increases in aortic SPAK and NKCC1 phosphorylation and attenuates angiotensin II–induced hypertension and contractile responses to angiotensin II in mesenteric arteries, while preserving basal myogenic tone [157]. Collectively, these findings demonstrate that WNK1, WNK3, and WNK4 contribute to VSMC function through distinct downstream pathways, including SPAK/NKCC1-dependent ion transport and TRPC3-mediated Ca^2+^ signaling (Figure 4).

In addition to contractility, WNK1 contributes to VSMC biology through regulation of vascular remodeling. Angiotensin II–induced vascular remodeling involves activation of the WNK1–FOXO3a signaling axis, leading to increased expression of matrix metalloproteinases MMP-2 and MMP-9, which promote extracellular matrix remodeling and may contribute to vascular hypertrophy and stiffness [158]. Thus, WNK1 in VSMCs supports at least two functionally distinct processes: SPAK/NKCC1-dependent regulation of contractility and FOXO3a-dependent control of matrix remodeling.

Evidence supporting a role for WNK2 in VSMCs remains limited. Unlike WNK1, WNK3, and WNK4, a defined role for WNK2 in vascular smooth muscle has not been established [80,99]. Together, these findings indicate that WNK1, WNK3, and WNK4 represent the major WNK isoforms with established roles in VSMC function, whereas the contribution of WNK2 remains poorly defined.

## WNKs, insulin signaling, and energy homeostasis

Multiple mechanisms connect WNKs to insulin action that can be reflected in metabolic and blood pressure disturbances. Elevated WNK function can result in impaired glucose homeostasis, exacerbating metabolic conditions such as diabetes and obesity. Several studies from the Uchida lab demonstrated that hyperinsulinemia activates WNK–OSR1/SPAK signaling, leading to increased NCC phosphorylation in db/db mice, a model for diabetes and obesity [159]. NCC activation results in enhanced sodium reabsorption, implicating WNKs in the dysregulation of salt balance common in metabolic disorders. Observations from a rat model also suggest an action of insulin on NCC and blood pressure [160].

WNK kinases interact with PI3K/AKT pathway, critical for insulin signaling, to facilitate glucose uptake. The first elements of the mechanism were uncovered by the Jordan lab, who showed that WNKs influence trafficking of glucose transporters to the plasma membrane by regulating the AKT substrate Rab GTPase activating protein AS160 (TBC1D4)–Rab8A complex [161]. In the central nervous system, WNKs influence glucose uptake by modifying GLUT4 (facilitated glucose transporter member 4) trafficking, essential for insulin-mediated glucose uptake [162]. WNK1 had been previously shown to be essential for insulin-stimulated GLUT4 trafficking in skeletal muscle [163]. WNKs have also been implicated in the regulation of adipocyte differentiation, further highlighting their dual role in renal function and energy metabolism in adipose tissue. The PI3K/AKT pathway has been shown to promote activation of the WNK cascade in hyperinsulinemic db/db mice [159]. Therefore, insulin could potentially regulate WNK1 function via AKT, although how it does so remains obscure. Although often referred to as activating in the literature [139,159], phosphorylation by AKT3 has been shown to induce WNK1 degradation in adipocytes to reduce diet-induced obesity [164]; these results may imply a regulatory step coupled to a negative feedback event. A study from our group showed that WNKs have an inhibitory effect on AKT activity [162]. This suggests a complex, context-specific inter-regulation between WNKs and AKT. WNK4 deletion in mice ameliorated diet-induced obesity by reducing expression of adipogenic genes, e.g., PPARγ, which is central to fat cell development [165–167].

Hyperglycemia and hyperinsulinemia result in excessive neddylation-dependent degradation of CUL3 adaptor proteins (KLHL2/3 and RhoBTB1) in the aorta and kidneys [168]. The resulting loss of adaptors impairs CRL3 E3 ligase activity, leading to the accumulation of multiple substrates, including WNK3 and RhoA. Elevated WNK3 in the aorta activates OSR1/SPAK–NKCC1 signaling, thereby enhancing vasoconstriction and sodium reabsorption [169]. Concurrently, this signaling cascade cooperates with the RhoA/ROCK pathway to synergistically increase vasoconstrictor sensitivity in diabetes. Increased WNK4 further augments sodium reabsorption, contributing to dysregulated vascular tone and sodium homeostasis under diabetic conditions.

## WNKs and the brain

Communication between WNKs and insulin pathways also extends to parts of the brain. WNKs regulate mouse behavior and alter central nervous system glucose uptake, notably in the hippocampus, where insulin sensitivity directly correlates with cognitive functions [162]. Through OSR1/SPAK, WNKs can influence two steps in the membrane trafficking pathway that directs GLUT4 to the plasma membrane in response to insulin. OSR1/SPAK binds AS160 (*TBC1D4*), which facilitates trafficking of GLUT4 to the membrane. OSR1/SPAK also binds to sortilin, which assists in assembly of insulin-responsive vesicles. These results illustrate that inhibition of WNKs enhances insulin signaling in the hippocampus, an area of the brain critical for memory and learning [162]. Beyond metabolic regulation, WNK1 also contributes to homeostatic sensing in the central nervous system. In OVLT osmosensory neurons, hypertonic stimulation activates WNK1, increasing neuronal excitability and promoting arginine vasopressin (AVP) release, thereby contributing to water balance and plasma osmolality control [170,171]. These findings demonstrate a connection between WNK kinase activity, insulin signaling, and glucose metabolism in the brain, implicating WNKs as potential contributors to metabolic regulation in neurodegenerative and neurodevelopmental conditions. Together, these findings underscore the broader role of WNK1 in linking neuronal activity to systemic physiological responses.

WNK kinases play a multifaceted role in modulating GABA (gamma-aminobutyric acid) neurotransmission through their regulation of chloride homeostasis via NKCC1 and KCC2. NKCC1 activation promotes chloride influx, which has been linked to neuronal depolarization and increased excitability, while KCC2 activity contributes to chloride efflux, typically promoting hyperpolarization [172,173]. GABA acts as the primary inhibitory neurotransmitter in the central nervous system, and its function is critically dependent on the intracellular concentration of chloride ions [174,175]. The opposing functions of NKCC1 and KCC2 create an essential mechanism where the directionality of GABA's action can switch from inhibitory to excitatory depending on their relative activities [86,176–179]. While the functions are most often associated with WNK1 and WNK3, WNK2 has also been shown to promote neuronal depolarization when GABA receptors are activated [180]. Yang et al*.* demonstrated that NO modulates WNK–OSR1/SPAK–NKCC1 signaling in serotonergic neurons, influencing GABAergic inhibition through chloride equilibrium [181].

WNKs impact various neurological conditions via control of chloride homeostasis, including epilepsy, where altered GABAergic signaling is often observed due to a dysregulation of the chloride transport machinery [175,182,183]. Elevated intracellular Cl^−^ can lead to hyperexcitability, a hallmark of epileptic activity [181]. Abnormalities in chloride homeostasis are evident in neurodevelopmental disorders, where synaptic dysfunctions may contribute to conditions such as autism or schizophrenia [179,184,185]. The intricate regulatory mechanisms involving WNK kinases underscore the potential for targeted therapeutic strategies in conditions characterized by altered GABA signaling, including epilepsy and other neurodevelopmental disorders.

Elvira *et al.* noted that OSR1 and SPAK also regulate large-conductance calcium-activated potassium (BK) channels, suggesting that these kinases modulate the excitability and dynamics of neuronal electrical activity by potentially impacting the repolarization phase of action potentials after depolarization [186]. Additionally, their involvement in regulating excitatory amino acid transporters (EAATs), such as EAAT1 and EAAT2, as shown in Xenopus oocytes, highlights how disturbances in their function could contribute to the neurotoxicity commonly observed in excitotoxic conditions [187].

Synaptic plasticity is essential for learning and memory. WNK1 and WNK4 have been found to induce the expression of LIM homeobox 8 (Lhx8), critical for cholinergic neuronal functions and the specification of neurons during development [188]. This suggests that WNKs may also affect the architecture of the developing nervous system.

WNK1 and WNK3 control NKCC1 during stresses including ischemia [189,190]. Elevated intracellular chloride due to high NKCC1 leads to detrimental cellular effects and contributes to neuronal cell death in stroke models [173]. Increased WNK3–SPAK activity has been associated with exacerbating brain damage following ischemic events [191,192]. Inhibition of this pathway presents a therapeutic approach to mitigate neuronal injury and promote recovery post-stroke [173,189,191]. The interplay between the WNK–SPAK–NKCC pathway and other signaling cascades, notably the NF-κB pathway—known for its roles in inflammation and cell survival—further complicates the clinical landscape of stroke pathophysiology [192]. Activation of NF-κB can enhance the expression of WNK kinases during ischemic stress, leading to downstream effects that may heighten neuronal apoptosis and alter inflammatory responses in the brain, creating a double-edged effect [192]. WNK1 expression was significantly reduced in subarachnoid hemorrhage patient blood and brain tissue, correlating negatively with microglial activation. WNK1 overexpression alleviated brain edema, neuronal necrosis, behavioral deficits, and inflammation by inhibiting NLRP3 inflammasome activation [193,194].

Inhibition of WNK1/FOXO3a/MMP signaling pathway was observed in a vascular smooth muscle-specific knockout of the volume-regulated Cl^−^ channel LRRC8A (leucine-rich repeat-containing protein 8A), which was protective against angiotensin II-induced cerebrovascular remodeling during hypertension [158]. In addition, WNK1 contributes to LRRC8A-induced blood–brain barrier damage post-ischemic stroke. These studies together suggest WNK1 to be an important target in treating stroke [195].

Investigating these interactions has led to the identification of WNK signaling as a potential biomarker for assessing stroke severity and prognosis. The activation of WNK pathways in response to ischemic events prompts investigations into genetic variations and expressions of WNK isoforms that could affect individual susceptibility to stroke and recovery [196]. By elucidating these mechanisms, it may become feasible to classify stroke patients based on their WNK signaling profiles and tailor treatments accordingly.

## WNK1 in pain sensing

Alternative splicing or mutations in the WNK1 nervous system-specific exon (hereditary sensory neuropathy type II (HSN2)) cause hereditary sensory and autonomic neuropathy type II (HSANII) [3,197]. This exon first uncovered the importance of WNK1 in neuronal function, particularly in sensory neuron development and the modulation of neuronal excitability. Various studies highlight HSN2’s impact on neuronal development, ion transport regulation, and neuropathic pain and hereditary sensory neuropathy. Shekarabi *et al.* provided insights into how mutations in the WNK1 HSN2 exon, which lead to loss of WNK1 functions, lead to impaired peripheral sensory perception, a hallmark of HSAN II [3]. They found that the expression of the WNK1 isoform containing HSN2 is notably more prevalent in sensory neurons than in motor neurons or elsewhere in the body, emphasizing its specialized function in sensory neuron integrity and nerve function. Additionally, mutations in this exon have been linked to various phenotypic manifestations, including congenital insensitivity to pain and early-onset sensory neuropathy [198,199]. Shimizu and Shibuya demonstrated that this exon modulates neural development via the activation of OSR1 and downstream pathways involving GSK3β, both of which are crucial for proper neuronal morphology [188].

The WNK1 isoform containing HSN2 also regulates chloride homeostasis through its interaction with KCC2, a transporter crucial for maintaining inhibitory neurotransmission in the central nervous system, as described above, increasing neuronal excitability and minimizing GABAergic inhibition in neuropathic pain models [200]. Studies by Bercier et al. noted that WNK1 with HSN2 mutations led to deregulated KCC2 expression, demonstrating a direct correlation between HSN2 functionality and the transcriptional regulation of KCC2, thereby affecting neuronal excitability and sensory nerve development [197]. This pathway contributes to the post-injury response mechanisms implicated in neuropathic pain, further illustrating the critical function of this WNK1 isoform in neurobiology and pain pathogenesis.

## WNKs in cancer

All four WNKs have been implicated in cancers, with WNK3 in glioma and WNK1 in breast cancer supported by more than a decade of evidence [201]. WNK1 was implicated from a transposon insertional mutagenesis study in mice that correlated data from over 2000 breast cancer patients [202]. Numerous other analyses of tumor genetics and linkage to drug resistance and severity place WNK1 on very short lists of predictive or prognostic markers, for example, a five-gene-based prognostic model for relapsed/refractory acute lymphoblastic leukemia [203]. What is lacking from most of these studies is specific or comparative mechanisms. Impact on immune cell function is sometimes indicated but is not described in detail here (e.g. [89,204]). Cancer genome sequencing studies have identified mutations in WNK family members, as well as gene fusions such as WNK1–B4GALNT3 [205,206], but whether these alterations are sufficient to drive cellular transformation on their own has not been directly tested; most of the mechanistic evidence discussed below instead reflects dysregulated WNK expression or activity rather than mutation.

The angiogenic properties of WNKs can contribute to tumorigenesis. As tumors grow, they release growth factors that stimulate blood vessel formation, providing oxygen and nutrients necessary for expansion [207,208]. This process also helps cancer cells spread to other parts of the body, driving metastasis. Multiple lines of evidence demonstrate a critical function of WNK1 in tumor angiogenesis. In a zebrafish human hepatoma xenograft model, endothelial WNK1 and OSR1 are up-regulated during tumor-induced angiogenesis, and genetic or pharmacologic inhibition of WNK1 suppresses ectopic vessel formation and limits tumor proliferation [209]. Conversely, endothelial-specific WNK1 overexpression accelerates tumor growth and metastatic spread [210]. These findings highlight WNK1 as a pro-angiogenic signaling node that supports tumor expansion.

Dysregulation of transport of ions and amino acids can also promote oncogenesis. WNKs 1 and 3 have been implicated in gliomas. The LRRC8A-containing chloride channel is crucial for cell volume recovery and survival under hypertonic conditions, mediating Cl^−^ efflux that activates the WNK/NKCC1 pathway and supports RVI under hypertonic stress [211]. An additional study on LRRC8 found a link of WNK1 to stimulation of mTORC2 and its downstream kinases AKT and SGK. Down-regulation of WNK1 or pharmacological inhibition of mTOR or SGK/AKT reduced glioblastoma proliferation much like LRRC8A knockdown [212]. In *in vitro* and *in vivo* mouse models of acute myeloid leukemia (AML), genetic depletion or pharmacological inhibition (using WNK463 or Compound 12) of WNK1 or its downstream kinases OSR1/SPAK suppressed proliferation and caused apoptosis [213]. Interestingly, in these models WNK1 increased mTORC1 activity by increasing amino acid uptake via SLC38A2 and other amino acid transporters. SLC38A2 bound to OSR1 and its phosphorylation was reduced by WNK inhibition, indicating that it is a direct target of the pathway and identifying another mechanism linking WNK1 to mTORC1 [213].

WNK1 regulation by microRNAs appears to be an emerging theme in cancer progression. Multiple miRNA-mediated pathways converge on WNK1 to control angiogenic and migratory phenotypes. miR-524-5p in colon cancer exemplifies this principle, as miR-524-5p directly suppresses WNK1 expression and its overexpression inhibits tumor-induced angiogenesis in HUVECs [214]. A similar mechanism is observed in hepatocellular carcinoma (HCC), where the long noncoding RNA LEF1-AS1 prevents miR-136-5p from repressing WNK1. Relief of suppression enhances HCC cell proliferation, migration, and angiogenesis, whereas LEF1-AS1 silencing reduces tumor growth and angiogenic markers such as MMP-9 and VEGF [215]. Interestingly, one of the predicted target genes of miR-136-5p is TRIM27, an E3 ubiquitin ligase and a member of the tripartite motif (TRIM) family. MiR-136-5p inhibits TRIM27 expression and exerts antitumor effects in HCC by reducing mesenchymal markers vimentin and N-cadherin while increasing the epithelial marker E-cadherin, suggesting its role in suppressing epithelial-mesenchymal transition (EMT) [216]. Knockdown of WNK1 disrupts the interaction between TRIM27 and its deubiquitinating enzyme USP7, causing reduced TRIM27 protein levels [217]. These findings suggest a possible mechanistic connection between miR-136-5p and WNK1–TRIM27, contributing to the inhibition of EMT, angiogenesis, and suppression of tumor progression in HCC. Another miRNA target prediction study identified WNK1 as a potential target of miR-93-5p, whose elevated expression in MDA-MB-231 triple-negative breast cancer cells reduced WNK1 protein and concomitantly decreased migration and invasion while increasing adhesion [218]. Consistent with these reports, WNK1 knockdown or pharmacological inhibition using WNK463 effectively suppressed cell migration and invasion [219,220], supporting the notion that multiple miRNA pathways regulate WNK1 to modulate both angiogenic and metastatic behaviors.

WNK1 dysregulation drives gastric cancer progression through multiple pathways. Phosphoproteomics revealed WNK1 hyperphosphorylation in diffuse gastric cancer [221]. The zinc-binding phosphoprotein ZYX promotes WNK1 activation, which up-regulates the transcription factor SNAI1 (Snail) to increase EMT and cell mobility in gastric cancer cells. WNK1 knockdown or pharmacological inhibition with WNK463 reverses these effects [222]. Phosphoglycerate dehydrogenase (PHGDH) is the rate-limiting enzyme in *de novo* serine biosynthesis and generates intermediates supporting cancer cell proliferation. In gastric cancer cell lines, PHGDH expression is increased, causing dysregulation of serine metabolism. WNK1 binds PHGDH and phosphorylates Ser349 and Ser371 to block PHGDH degradation and increase its activity. Mutation of these sites or inhibition of WNK1 decreased PHGDH as well as tumor cell proliferation [223].

WNK1 is up-regulated in HCC, where its expression correlates with poor prognosis, competing with NRF2 for KEAP1 binding to enhance NRF2 nuclear accumulation and antioxidant signaling [224], a regulatory relationship similarly observed in pancreatic cancer [225]. WNK1 activity is elevated in lenvatinib-resistant HCC cells, with FAK promoting this resistance by modulating WNK1 activity. Pharmacological inhibition of WNK1 with WNK463 restores lenvatinib sensitivity, whereas WNK1 overexpression diminishes drug responsiveness [226]. WNK1 also enhances glycolytic metabolism in HCC. Ubiquitination and degradation of WNK1 by the E3 ligase SIAH2 limit glycolysis and malignant phenotypes, whereas WNK1 accumulation drives proliferation and stemness [227]. Similar metabolic reprogramming occurs in ovarian cancer, where WNK1 up-regulation increases GLUT1 and glycolytic enzymes (LDHA, PKM2, PDK1) and HIF-1α expression, promoting tumor growth and metastasis [228]. WNK1 is also hyperphosphorylated in ovarian cancer and activates the MEK5-ERK5 pathway, a driver of tumor proliferation [229].

Recently, WNK1 has been identified as a critical regulator of MYC expression in multiple myeloma, where its inhibition suppresses cell growth and induces cell-cycle arrest, senescence, and apoptosis, and where WNK463 suppresses tumor growth in both primary patient samples and xenograft models [230]. Additionally, WNK1 kinase activity contributes to AKT-mediated chemoresistance in multiple myeloma [231]. Similarly, in glioblastoma, WNK1 is thought to link connexin 43 (Cx43) to c-MYC, promoting proliferation, survival, and tumor initiation [232]. The polycomb repressor complex component EZH2 represses KCC2, which is associated with activation of WNK1/OSR1/NKCC1 signaling and tumor progression, whereas EZH2 inhibition reduces WNK1 activity and suppresses glioma cell migration and invasion [233]. In other cancer types, WNK1 has been proposed as a diagnostic biomarker in cholangiocarcinoma and is thought to drive tumor progression by enhancing neutrophil infiltration and angiogenesis through CXCL5- and VEGFA-mediated signaling [234]. Elevated WNK1 expression correlates with poor survival and adverse clinicopathological features in patients with colorectal adenocarcinoma [235], and urine exosome proteomics identified WNK1 as a protein that increases with age and is also elevated in lung cancer, suggesting its potential utility in a diagnostic biomarker panel [236]. Pleiotropic analyses further implicate WNK1 in the genetic overlap between esophageal and lung carcinomas, pointing to shared regulatory mechanisms [237]. These findings support WNK1 as a potential prognostic marker and therapeutic target across diverse cancer types.

WNK1 influences both actin and microtubule dynamics in ways that can modulate cancer progression. In breast cancer cells, WNK1 regulates endosomal actin polymerization and receptor trafficking via the TRIM27-USP7 complex, thereby limiting degradation of the receptor tyrosine kinases EGFR and AXL and enhancing invasive potential [217]. WNK1 also regulates microtubule polymerization [238]. In malignant pleural mesothelioma, it functions as a key microtubule regulatory protein, with microtubule-targeting drugs (e.g., CIT-026 and CIT-223) inducing WNK1 phosphorylation that leads to aberrant spindle formation, mitotic arrest, and apoptosis [239]. Additionally, WNK1 depletion alters cellular responses to mitotic inhibitors like rigosertib and increases sensitivity to microtubule-stabilizing agents such as paclitaxel, underscoring its role in microtubule dynamics and chemotherapy response [240], suggesting that targeting WNK signaling could modulate cytoskeletal regulation to improve therapeutic outcomes.

The accumulated findings provide strong evidence that WNK can function as a contributor to tumor progression by regulating essential processes in various cancers. The complexity of its actions is often context-dependent. Specific WNK inhibitors may hold promise for cancer treatment, but further research is needed to fully understand their mechanisms of action relevant to each cancer type and assess their safety and efficacy in clinical settings.

## Conclusions

WNKs are ancient kinases that have been adapted to supervise a surprising number of cellular pathways and functions. WNKs control ion transport through both positive and negative actions on OSR1/SPAK-sensitive ion cotransporters and the context-dependent stabilization and membrane localization of a large number of other transporters and ion channels, thereby determining set points essential for kidney function, blood pressure, and neurotransmission. Their broader impact on metabolic processes through associations with mTOR and PI3K pathway components and transporters for amino acids and metabolites is becoming more evident, and their versatility has been exploited by numerous cancers. WNK versatility arises in part from the capacity to be activated in both high and low osmotic conditions due to sensitivity to concentrations of ions and small molecules, their many interacting partners, their regulation in condensates, and also their susceptibility to many degradative mechanisms.

## Supplementary Material

Supplementary Table S1

## Open Access

Open access for this article was enabled by the participation of the University of Texas Southwestern Medical Center in an all-inclusive Read & Publish agreement with Portland Press and the Biochemical Society.

## Abbreviations

AML: acute myeloid leukemia
AVP: arginine vasopressin
Cab39: calcium-binding protein 39
CCT: conserved C-terminal
CRL3: cullin3 ring ubiquitin ligase complexes
CUL3: cullin3
EAAT: excitatory amino acid transporter
ENaC: epithelial Na^+^ channel
FHHt: familial hyperkalemic hypertension
GABA: gamma-aminobutyric acid (neurotransmitter)
GLUT4: facilitated glucose transporter member 4
HCC: hepatocellular carcinoma
HSANII: hereditary sensory and autonomic neuropathy type II
HSN2: hereditary sensory neuropathy type II
HUVEC: human umbilical vein endothelial cells
KCC: K^+^ Cl^−^ cotransporter
Kir/IRK: inward rectifier K+ channel
KLHL3: kelch-like protein 3
KS-WNK1: kidney specific (isoform) WNK1
L-WNK1: long (isoform) WNK1
Lhx8: LIM homeobox 8
LRRC8A: leucine-rich repeat-containing protein 8A (volume-regulated anion channel subunit)
mTORC: mammalian target of rapamycin complex
NCC: Na^+^ Cl^-^ cotransporter
NKCC: Na^+^ K^+^ 2Cl^-^ cotransporter
NO: nitric oxide
NRBP: nuclear receptor-binding protein
OSR1: oxidative stress-responsive kinase 1
PHAII: pseudohypoaldosteronism type II
PHGDH: phosphoglycerate dehydrogenase
PI3K: phosphoinositide 3-kinase
PPAR: peroxisome proliferator-activated receptor
RVD: regulatory volume decrease
RVI: regulatory volume increase
SGK1: serum/glucocorticoid-regulated kinase 1
SPAK: SPS1-related proline/alanine-rich kinase
TBC1D4: TBC1 domain family member 4
TGF-β: transforming growth factor-β
TRPV: transient receptor potential cation channel subfamily V
TSC22D: TSC22 domain family protein
VEGF: vascular endothelial growth factor
VEGFR2: vascular endothelial growth factor receptor 2
VSMCs: vascular smooth muscle cells
WNK: with no lysine (K)

## References

[1] Wilson F.H., Disse-Nicodeme S., Choate K.A., Ishikawa K., Nelson-Williams C., Desitter I. et al. (2001) Human hypertension caused by mutations in WNK kinases. Science 293, 1107–1112 11498583 10.1126/science.1062844

[2] Xie J., Wu T., Xu K., Huang I.K., Cleaver O. and Huang C.L. (2009) Endothelial-specific expression of WNK1 kinase is essential for angiogenesis and heart development in mice. Am. J. Pathol. 175, 1315–1327 19644017 10.2353/ajpath.2009.090094 PMC2731149

[3] Shekarabi M., Girard N., Riviere J.B., Dion P., Houle M., Toulouse A. et al. (2008) Mutations in the nervous system–specific HSN2 exon of WNK1 cause hereditary sensory neuropathy type II. J. Clin. Invest. 118, 2496–2505 18521183 10.1172/JCI34088 PMC2398735

[4] Xu B., English J.M., Wilsbacher J.L., Stippec S., Goldsmith E.J. and Cobb M.H. (2000) WNK1, a novel mammalian serine/threonine protein kinase lacking the catalytic lysine in subdomain II. J. Biol. Chem. 275, 16795–16801 10828064 10.1074/jbc.275.22.16795

[5] Yamada K., Levell J., Yoon T., Kohls D., Yowe D., Rigel D.F. et al. (2017) Optimization of allosteric with-no-lysine (WNK) kinase inhibitors and efficacy in rodent hypertension models. J. Med. Chem. 60, 7099–7107 28771350 10.1021/acs.jmedchem.7b00708

[6] Yamada K., Park H.M., Rigel D.F., DiPetrillo K., Whalen E.J., Anisowicz A. et al. (2016) Small-molecule WNK inhibition regulates cardiovascular and renal function. Nat. Chem. Biol. 12, 896–898 27595330 10.1038/nchembio.2168

[7] Chlebowicz J., Akella R., Humphreys J.M., He H., Kannangara A.R., Wei S. et al. (2023) Identification of a class of WNK isoform-specific inhibitors through high-throughput screening. Drug Des. Devel. Ther. 17, 93–105 36712947 10.2147/DDDT.S389461 PMC9880028

[8] Boyd-Shiwarski C.R., Shiwarski D.J., Griffiths S.E., Beacham R.T., Norrell L., Morrison D.E. et al. (2022) WNK kinases sense molecular crowding and rescue cell volume via phase separation. Cell 185, 4488.e4420–4506.e4420 36318922 10.1016/j.cell.2022.09.042 PMC9699283

[9] Thastrup J.O., Rafiqi F.H., Vitari A.C., Pozo-Guisado E., Deak M., Mehellou Y. et al. (2012) SPAK/OSR1 regulate NKCC1 and WNK activity: analysis of WNK isoform interactions and activation by T-loop *trans*-autophosphorylation. Biochem. J. 441, 325–337 22032326 10.1042/BJ20111879 PMC3242505

[10] Bartual S.G., Pinkas D.M., Bufton J.C., Kupinska K., Wang D., Chalk R. et al. (2017) Crystal structure of WNK3 kinase and CCT1 didomain in a unphosphorylated state (5O2C). Protein Data Bank

[11] Moon T.M., Correa F., Kinch L.N., Piala A.T., Gardner K.H. and Goldsmith E.J. (2013) Solution structure of the WNK1 autoinhibitory domain, a WNK-specific PF2 domain. J. Mol. Biol. 425, 1245–1252 23376100 10.1016/j.jmb.2013.01.031

[12] Pinkas D.M., Bufton J.C., Burgess-Brown N.A., Arrowsmith C.H., Edwards A.M., Bountra C. et al. (2017) Crystal structure of the human WNK2 CCT-like 1 domain in complex with a WNK1 RFXV peptide (6FBK). Protein Data Bank 10.2210/pdb6elm/pdb

[13] Taylor C.A. and Cobb M.H. (2022) CCT and CCT-like modular protein interaction domains in WNK signaling. Mol. Pharmacol. 101, 201 34312216 10.1124/molpharm.121.000307 PMC9092477

[14] Xu B.E., Min X., Stippec S., Lee B.H., Goldsmith E.J. and Cobb M.H. (2002) Regulation of WNK1 by an autoinhibitory domain and autophosphorylation. J. Biol. Chem. 277, 48456–48462 12374799 10.1074/jbc.M207917200

[15] Elvers K.T., Lipka-Lloyd M., Trueman R.C., Bax B.D. and Mehellou Y. (2022) Structures of the human SPAK and OSR1 conserved C-terminal (CCT) domains. ChemBioChem 23, e202100441 34726826 10.1002/cbic.202100441

[16] Piechotta K., Garbarini N., England R. and Delpire E. (2003) Characterization of the interaction of the stress kinase SPAK with the Na^+^-K^+^-2Cl^-^ cotransporter in the nervous system: evidence for a scaffolding role of the kinase. J. Biol. Chem. 278, 52848–52856 14563843 10.1074/jbc.M309436200

[17] Piechotta K., Lu J. and Delpire E. (2002) Cation chloride cotransporters interact with the stress-related kinases Ste20-related proline-alanine-rich kinase (SPAK) and oxidative stress response 1 (OSR1). J. Biol. Chem. 277, 50812–50819 12386165 10.1074/jbc.M208108200

[18] Villa F., Goebel J., Rafiqi F.H., Deak M., Thastrup J., Alessi D.R. et al. (2007) Structural insights into the recognition of substrates and activators by the OSR1 kinase. EMBO Rep. 8, 839–845 17721439 10.1038/sj.embor.7401048 PMC1973955

[19] Taylor C.A. 4th, An S.W., Kankanamalage S.G., Stippec S., Earnest S., Trivedi A.T. et al. (2018) OSR1 regulates a subset of inward rectifier potassium channels via a binding motif variant. Proc. Natl. Acad. Sci. U.S.A. 115, 3840–3845 29581290 10.1073/pnas.1802339115 PMC5899495

[20] Taylor C.A. 4th, Jung J.U., Gallolu Kankanamalage S., Li J., Grzemska M., Jaykumar A.B. et al. (2025) Kinase interaction analysis predicts actions of the WNK-OSR1/SPAK pathway. Commun. Biol. 8, 1335 40913030 10.1038/s42003-025-08551-5 PMC12413468

[21] Jumper J., Evans R., Pritzel A., Green T., Figurnov M., Ronneberger O. et al. (2021) Highly accurate protein structure prediction with AlphaFold. Nature 596, 583–589 34265844 10.1038/s41586-021-03819-2 PMC8371605

[22] Varadi M., Anyango S., Deshpande M., Nair S., Natassia C., Yordanova G. et al. (2021) AlphaFold Protein Structure Database: massively expanding the structural coverage of protein-sequence space with high-accuracy models. Nucleic Acids Res. 50, D439–D444 10.1093/nar/gkab1061 PMC8728224 34791371

[23] Moriguchi T., Urushiyama S., Hisamoto N., Iemura S., Uchida S., Natsume T. et al. (2005) WNK1 regulates phosphorylation of cation-chloride-coupled cotransporters via the STE20-related kinases, SPAK and OSR1. J. Biol. Chem. 280, 42685–42693 16263722 10.1074/jbc.M510042200

[24] Sengupta S., Tu S.W., Wedin K., Earnest S., Stippec S., Luby-Phelps K. et al. (2012) Interactions with WNK (with no lysine) family members regulate oxidative stress response 1 and ion co-transporter activity. J. Biol. Chem. 287, 37868–37879 22989884 10.1074/jbc.M112.398750 PMC3488059

[25] Rodriguez M., Kannangara A., Chlebowicz J., Akella R., He H., Tambar U.K. et al. (2022) Synthesis and structural characterization of novel trihalo-sulfone inhibitors of WNK1. ACS Med. Chem. Lett. 13, 1678–1684 36262391 10.1021/acsmedchemlett.2c00216 PMC9575160

[26] Yamada K., Zhang J.-H., Xie X., Reinhardt J., Xie A.Q., LaSala D. et al. (2016) Discovery and characterization of allosteric WNK kinase inhibitors. ACS Chem. Biol. 11, 3338–3346 27712055 10.1021/acschembio.6b00511

[27] AlAmri M.A., Kadri H., Alderwick L.J., Simpkins N.S. and Mehellou Y. (2017) Rafoxanide and closantel inhibit SPAK and OSR1 kinases by binding to a highly conserved allosteric site on their C-terminal domains. ChemMedChem 12, 639–645 28371477 10.1002/cmdc.201700077

[28] Kikuchi E., Mori T., Zeniya M., Isobe K., Ishigami-Yuasa M., Fujii S. et al. (2015) Discovery of novel SPAK inhibitors that block WNK kinase signaling to cation chloride transporters. J. Am. Soc. Nephrol. 26, 1525–1536 25377078 10.1681/ASN.2014060560 PMC4483590

[29] Mori T., Kikuchi E., Watanabe Y., Fujii S., Ishigami-Yuasa M., Kagechika H. et al. (2013) Chemical library screening for WNK signaling inhibitors by using fluorescent correlation spectroscopy. Biochem. J. 455, 339–345 23981180 10.1042/BJ20130597

[30] Zhang J., Bhuiyan M.I.H., Zhang T., Karimy J.K., Wu Z., Fiesler V.M. et al. (2020) Modulation of brain cation-Cl(-) cotransport via the SPAK kinase inhibitor ZT-1a. Nat. Commun. 11, 78 31911626 10.1038/s41467-019-13851-6 PMC6946680

[31] Piala A.T., Moon T.M., Akella R., He H., Cobb M.H. and Goldsmith E.J. (2014) Chloride sensing by WNK1 involves inhibition of autophosphorylation. Sci. Signal. 7, ra41 24803536 10.1126/scisignal.2005050 PMC4123527

[32] Taylor C.A., Juang Y.C., Earnest S., Sengupta S., Goldsmith E.J. and Cobb M.H. (2015) Domain-swapping switch point in Ste20 protein kinase SPAK. Biochemistry 54, 5063–5071 26208601 10.1021/acs.biochem.5b00593 PMC5167558

[33] Lee S.J., Cobb M.H. and Goldsmith E.J. (2009) Crystal structure of domain-swapped STE20 OSR1 kinase domain. Protein Sci. 18, 304–313 19177573 10.1002/pro.27 PMC2708061

[34] Villa F., Deak M., Alessi D.R. and van Aalten D.M. (2008) Structure of the OSR1 kinase, a hypertension drug target. Proteins 73, 1082–1087 18831043 10.1002/prot.22238

[35] Ferdaus M.Z., Terker A.S., Koumangoye R.B., Al-Qusairi L., Welling P.A. and Delpire E. (2024) Deletion of KS-WNK1 promotes NCC activation by increasing WNK1/4 abundance. Am. J. Physiol. Renal. Physiol. 327, F373–F385 38961847 10.1152/ajprenal.00101.2024 PMC11460338

[36] Delaloy C., Lu J., Houot A.M., Disse-Nicodeme S., Gasc J.M., Corvol P. et al. (2003) Multiple promoters in the WNK1 gene: one controls expression of a kidney-specific kinase-defective isoform. Mol. Cell. Biol. 23, 9208–9221 14645531 10.1128/MCB.23.24.9208-9221.2003 PMC309643

[37] Kahle K.T., Rinehart J., de Los Heros P., Louvi A., Meade P., Vazquez N. et al. (2005) WNK3 modulates transport of Cl- in and out of cells: implications for control of cell volume and neuronal excitability. Proc. Natl. Acad. Sci. U.S.A. 102, 16783–16788 16275911 10.1073/pnas.0508307102 PMC1283843

[38] Lenertz L.Y., Lee B.H., Min X., Xu B.E., Wedin K., Earnest S. et al. (2005) Properties of WNK1 and implications for other family members. J. Biol. Chem. 280, 26653–26658 15883153 10.1074/jbc.M502598200

[39] Richardson C. and Alessi D.R. (2008) The regulation of salt transport and blood pressure by the WNK-SPAK/OSR1 signalling pathway. J. Cell Sci. 121, 3293–3304 18843116 10.1242/jcs.029223

[40] Zagorska A., Pozo-Guisado E., Boudeau J., Vitari A.C., Rafiqi F.H., Thastrup J. et al. (2007) Regulation of activity and localization of the WNK1 protein kinase by hyperosmotic stress. J. Cell Biol. 176, 89–100 17190791 10.1083/jcb.200605093 PMC2063630

[41] Akella R., Humphreys J.M., Sekulski K., He H., Durbacz M., Chakravarthy S. et al. (2021) Osmosensing by WNK kinases. Mol. Biol. Cell 32, mbcE20010089 10.1091/mbc.E20-01-0089 PMC8684725 33689398

[42] Min X., Lee B.H., Cobb M.H. and Goldsmith E.J. (2004) Crystal structure of the kinase domain of WNK1, a kinase that causes a hereditary form of hypertension. Structure 12, 1303–1311 15242606 10.1016/j.str.2004.04.014

[43] Akella R., Drozdz M.A., Humphreys J.M., Jiou J., Durbacz M.Z., Mohammed Z.J. et al. (2020) A phosphorylated intermediate in the activation of WNK kinases. Biochemistry 59, 1747–1755 32314908 10.1021/acs.biochem.0c00146 PMC7914002

[44] Teixeira L.R., Akella R., Humphreys J.M., He H. and Goldsmith E.J. (2024) Water and chloride as allosteric inhibitors in WNK kinase osmosensing. eLife 12, RP88224 39584807 10.7554/eLife.88224 PMC11588334

[45] Jung J.U., Stippec S. and Cobb M.H. (2025) Activation of WNK1 signaling through Piezo1. Proc. Natl. Acad. Sci. U.S.A. 122, e2513155122 40880539 10.1073/pnas.2513155122 PMC12415193

[46] Cornelius R.J., Maeoka Y., Shinde U. and McCormick J.A. (2024) Familial hyperkalemic hypertension. Compr. Physiol. 14, 5839–5874 39699086 10.1002/j.2040-4603.2024.tb00807.x PMC12327360

[47] Ohta A., Schumacher F.R., Mehellou Y., Johnson C., Knebel A., Macartney T.J. et al. (2013) The CUL3-KLHL3 E3 ligase complex mutated in Gordon's hypertension syndrome interacts with and ubiquitylates WNK isoforms: disease-causing mutations in KLHL3 and WNK4 disrupt interaction. Biochem. J. 451, 111–122 23387299 10.1042/BJ20121903 PMC3632089

[48] Takahashi D., Mori T., Wakabayashi M., Mori Y., Susa K., Zeniya M. et al. (2013) KLHL2 interacts with and ubiquitinates WNK kinases. Biochem. Biophys. Res. Commun. 437, 457–462 23838290 10.1016/j.bbrc.2013.06.104

[49] Zeniya M., Morimoto N., Takahashi D., Mori Y., Mori T., Ando F. et al. (2015) Kelch-like protein 2 mediates angiotensin ii-with no lysine 3 signaling in the regulation of vascular tonus. J. Am. Soc. Nephrol. 26, 2129–2138 25556166 10.1681/ASN.2014070639 PMC4552111

[50] Chen Z., Zhang J., Murillo-de-Ozores A.R., Castañeda-Bueno M., D'Amico F., Heilig R. et al. (2022) Sequence and structural variations determining the recruitment of WNK kinases to the KLHL3 E3 ligase. Biochem. J. 479, 661–675 35179207 10.1042/BCJ20220019 PMC9022995

[51] Louis-Dit-Picard H., Kouranti I., Rafael C., Loisel-Ferreira I., Chavez-Canales M., Abdel-Khalek W. et al. (2020) Mutation affecting the conserved acidic WNK1 motif causes inherited hyperkalemic hyperchloremic acidosis. J. Clin. Invest. 130, 6379–6394 32790646 10.1172/JCI94171 PMC7685730

[52] Hureaux M., Mazurkiewicz S., Boccio V., Vargas-Poussou R. and Jeunemaitre X. (2021) The variety of genetic defects explains the phenotypic heterogeneity of familial hyperkalemic hypertension. Kidney Int. Rep. 6, 2639–2652 34622103 10.1016/j.ekir.2021.07.025 PMC8484123

[53] Castaneda-Bueno M., Arroyo J.P., Zhang J., Puthumana J., Yarborough O. 3rd, Shibata S. et al. (2017) Phosphorylation by PKC and PKA regulate the kinase activity and downstream signaling of WNK4. Proc. Natl. Acad. Sci. U.S.A. 114, E879–E886 28096417 10.1073/pnas.1620315114 PMC5293014

[54] Yoshizaki Y., Mori Y., Tsuzaki Y., Mori T., Nomura N., Wakabayashi M. et al. (2015) Impaired degradation of WNK by Akt and PKA phosphorylation of KLHL3. Biochem. Biophys. Res. Commun. 467, 229–234 26435498 10.1016/j.bbrc.2015.09.184

[55] Ishizawa K., Wang Q., Li J., Xu N., Nemoto Y., Morimoto C. et al. (2019) Inhibition of sodium glucose cotransporter 2 attenuates the dysregulation of Kelch-Like 3 and NaCl cotransporter in obese diabetic mice. J. Am. Soc. Nephrol. 30, 782–794 30914436 10.1681/ASN.2018070703 PMC6493993

[56] Hu J., Huynh D.T., Dunn D.E., Wu J., Manriquez-Rodriguez C., Zhang Q.E. et al. (2025) Evidence for functional regulation of the KLHL3/WNK pathway by O-GlcNAcylation. Glycobiology 35, cwaf046 40796245 10.1093/glycob/cwaf046 PMC12360702

[57] Roy A., Al-Qusairi L., Donnelly B.F., Ronzaud C., Marciszyn A.L., Gong F. et al. (2015) Alternatively spliced proline-rich cassettes link WNK1 to aldosterone action. J. Clin. Invest. 125, 3433–3448 26241057 10.1172/JCI75245 PMC4588284

[58] Jaykumar A.B., Plumber S., Binns D., Wichaidit C., Luby-Phelps K. and Cobb M.H. (2024) SMURF1/2 are novel regulators of WNK1 stability. Kinases Phosphatases 2, 294–305 40895768 10.3390/kinasesphosphatases2030019 PMC12392294

[59] Jung J.U., Gosh A.B., Earnest S., Deaton S.L. and Cobb M.H. (2022) UBR5 is a novel regulator of WNK1 stability. Am. J. Physiol. Cell Physiol. 322, C1176–C1186 35442829 10.1152/ajpcell.00417.2021 PMC9169826

[60] Debonneville C., Flores S.Y., Kamynina E., Plant P.J., Tauxe C., Thomas M.A. et al. (2001) Phosphorylation of Nedd4-2 by Sgk1 regulates epithelial Na(+) channel cell surface expression. EMBO J. 20, 7052–7059 11742982 10.1093/emboj/20.24.7052 PMC125341

[61] Arroyo J.P., Lagnaz D., Ronzaud C., Vazquez N., Ko B.S., Moddes L. et al. (2011) Nedd4-2 modulates renal Na^+^-Cl^-^ cotransporter via the aldosterone-SGK1-Nedd4-2 pathway. J. Am. Soc. Nephrol. 22, 1707–1719 21852580 10.1681/ASN.2011020132 PMC3171941

[62] Wiemuth D., Lott J.S., Ly K., Ke Y., Teesdale-Spittle P., Snyder P.M. et al. (2010) Interaction of serum- and glucocorticoid regulated kinase 1 (SGK1) with the WW-domains of Nedd4-2 is required for epithelial sodium channel regulation. PLoS One 5, e12163 20730100 10.1371/journal.pone.0012163 PMC2921341

[63] Heise C.J., Xu B.E., Deaton S.L., Cha S.K., Cheng C.J., Earnest S. et al. (2010) Serum and glucocorticoid-induced kinase (SGK) 1 and the epithelial sodium channel are regulated by multiple with no lysine (WNK) family members. J. Biol. Chem. 285, 25161–25167 20525693 10.1074/jbc.M110.103432 PMC2919078

[64] Xu B.E., Stippec S., Chu P.Y., Lazrak A., Li X.J., Lee B.H. et al. (2005) WNK1 activates SGK1 to regulate the epithelial sodium channel. Proc. Natl. Acad. Sci. U.S.A. 102, 10315–10320 16006511 10.1073/pnas.0504422102 PMC1177404

[65] Xu B.E., Stippec S., Lazrak A., Huang C.L. and Cobb M.H. (2005) WNK1 activates SGK1 by a phosphatidylinositol 3-kinase-dependent and non-catalytic mechanism. J. Biol. Chem. 280, 34218–34223 16081417 10.1074/jbc.M505735200

[66] Saha B., Leite-Dellova D.C.A., Demko J., Sørensen M.V., Takagi E., Gleason C.E. et al. (2022) WNK1 is a chloride-stimulated scaffold that regulates mTORC2 activity and ion transport. J. Cell Sci. 135, jcs260313 36373794 10.1242/jcs.260313 PMC9789407

[67] Gagnon K.B. and Delpire E. (2012) Molecular physiology of SPAK and OSR1: two Ste20-related protein kinases regulating ion transport. Physiol. Rev. 92, 1577–1617 23073627 10.1152/physrev.00009.2012 PMC4519243

[68] Anselmo A.N., Earnest S., Chen W., Juang Y.C., Kim S.C., Zhao Y. et al. (2006) WNK1 and OSR1 regulate the Na^+^, K^+^, 2Cl^-^ cotransporter in HeLa cells. Proc. Natl. Acad. Sci. U.S.A. 103, 10883–10888 16832045 10.1073/pnas.0604607103 PMC1544143

[69] Vitari A.C., Deak M., Morrice N.A. and Alessi D.R. (2005) The WNK1 and WNK4 protein kinases that are mutated in Gordon’s hypertension syndrome phosphorylate and activate SPAK and OSR1 protein kinases. Biochem. J. 391, 17–24 16083423 10.1042/BJ20051180 PMC1237134

[70] Filippi B.M., de los Heros P., Mehellou Y., Navratilova I., Gourlay R., Deak M. et al. (2011) MO25 is a master regulator of SPAK/OSR1 and MST3/MST4/YSK1 protein kinases. EMBO J. 30, 1730–1741 21423148 10.1038/emboj.2011.78 PMC3101989

[71] Mehellou Y., Alamri M.A., Dhiani B.A. and Kadri H. (2018) C-terminal phosphorylation of SPAK and OSR1 kinases promotes their binding and activation by the scaffolding protein MO25. Biochem. Biophys. Res. Commun. 503, 1868–1873 30060950 10.1016/j.bbrc.2018.07.128

[72] Mehellou Y., Alessi D.R., Macartney T.J., Szklarz M., Knapp S. and Elkins J.M. (2013) Structural insights into the activation of MST3 by MO25. Biochem. Biophys. Res. Commun. 431, 604–609 23296203 10.1016/j.bbrc.2012.12.113 PMC3725419

[73] Shi Z., Jiao S., Zhang Z., Ma M., Zhang Z., Chen C. et al. (2013) Structure of the MST4 in complex with MO25 provides insights into its activation mechanism. Structure 21, 449–461 23434407 10.1016/j.str.2013.01.007

[74] Xiao Y.X., Lee S.Y., Aguilera-Uribe M., Samson R., Au A., Khanna Y. et al. (2024) The TSC22D, WNK, and NRBP gene families exhibit functional buffering and evolved with Metazoa for cell volume regulation. Cell Rep. 43, 114417 38980795 10.1016/j.celrep.2024.114417

[75] Grimm P.R., Taneja T.K., Liu J., Coleman R., Chen Y.Y., Delpire E. et al. (2012) SPAK isoforms and OSR1 regulate sodium-chloride co-transporters in a nephron-specific manner. J. Biol. Chem. 287, 37673–37690 22977235 10.1074/jbc.M112.402800 PMC3488044

[76] Sengupta S., Lorente-Rodriguez A., Earnest S., Stippec S., Guo X., Trudgian D.C. et al. (2013) Regulation of OSR1 and the sodium, potassium, two chloride cotransporter by convergent signals. Proc. Natl. Acad. Sci. U.S.A. 110, 18826–18831 24191005 10.1073/pnas.1318676110 PMC3839713

[77] Xu Y., Li C., Yin H., Nowsheen S., Xu X., Kang W. et al. (2024) STK39-mediated amplification of γ-H2A.X promotes homologous recombination and contributes to PARP inhibitor resistance. Nucleic Acids Res. 52, 13881–13895 39588777 10.1093/nar/gkae1099 PMC11662673

[78] Liu Z., Demian W., Persaud A., Jiang C., Subramanaya A.R. and Rotin D. (2022) Regulation of the p38-MAPK pathway by hyperosmolarity and by WNK kinases. Sci. Rep. 12, 14480 36008477 10.1038/s41598-022-18630-w PMC9411163

[79] Chen W., Yazicioglu M. and Cobb M.H. (2004) Characterization of OSR1, a member of the mammalian Ste20p/germinal center kinase subfamily. J. Biol. Chem. 279, 11129–11136 14707132 10.1074/jbc.M313562200

[80] Dbouk H.A., Weil L.M., Perera G.K., Dellinger M.T., Pearson G., Brekken R.A. et al. (2014) Actions of the protein kinase WNK1 on endothelial cells are differentially mediated by its substrate kinases OSR1 and SPAK. Proc. Natl. Acad. Sci. U.S.A. 111, 15999–16004 25362046 10.1073/pnas.1419057111 PMC4234555

[81] Flagella M., Clarke L.L., Miller M.L., Erway L.C., Giannella R.A., Andringa A. et al. (1999) Mice lacking the basolateral Na-K-2Cl cotransporter have impaired epithelial chloride secretion and are profoundly deaf. J. Biol. Chem. 274, 26946–26955 10480906 10.1074/jbc.274.38.26946

[82] Koumangoye R., Bastarache L. and Delpire E. (2021) NKCC1: newly found as a human disease-causing ion transporter. Function (Oxf) 2, zqaa028 33345190 10.1093/function/zqaa028 PMC7727275

[83] Kahle K.T., Khanna A.R., Alper S.L., Adragna N.C., Lauf P.K., Sun D. et al. (2015) K-Cl cotransporters, cell volume homeostasis, and neurological disease. Trends Mol. Med. 21, 513–523 26142773 10.1016/j.molmed.2015.05.008 PMC4834970

[84] Mercado A., de Los Heros P., Melo Z., Chavez-Canales M., Murillo-de-Ozores A.R., Moreno E. et al. (2016) With no lysine L-WNK1 isoforms are negative regulators of the K^+^-Cl^-^ cotransporters. Am. J. Physiol. Cell Physiol. 311, C54–C66 27170636 10.1152/ajpcell.00193.2015 PMC4967140

[85] Hartmann A.M. and Nothwang H.G. (2022) NKCC1 and KCC2: Structural insights into phospho-regulation. Front. Mol. Neurosci. 15, 964488 35935337 10.3389/fnmol.2022.964488 PMC9355526

[86] Kahle K.T., Rinehart J. and Lifton R.P. (2010) Phosphoregulation of the Na-K-2Cl and K-Cl cotransporters by the WNK kinases. Biochim. Biophys. Acta 1802, 1150–1158 20637866 10.1016/j.bbadis.2010.07.009 PMC3529164

[87] Furusho T., Uchida S. and Sohara E. (2020) The WNK signaling pathway and salt-sensitive hypertension. Hypertens. Res. 43, 733–743 32286498 10.1038/s41440-020-0437-x

[88] Ueda K. and Shimosawa T. (2024) Regulating distal nephron functions and salt sensitivity. Am. J. Physiol. Renal. Physiol. 327, F566–F580 39024355 10.1152/ajprenal.00103.2024

[89] Koumangoye R. (2022) The role of Cl(-) and K(+) efflux in NLRP3 inflammasome and innate immune response activation. Am. J. Physiol. Cell Physiol. 322, C645–C652 35171697 10.1152/ajpcell.00421.2021

[90] Haas B.R., Cuddapah V.A., Watkins S., Rohn K.J., Dy T.E. and Sontheimer H. (2011) With-No-Lysine Kinase 3 (WNK3) stimulates glioma invasion by regulating cell volume. Am. J. Physiol. Cell Physiol. 301, C1150–C1160 21813709 10.1152/ajpcell.00203.2011 PMC3213919

[91] Amnekar R.V., Dite T., Lis P., Bell S., Brown F., Johnson C. et al. (2025) NRBP1 pseudokinase binds to and activates the WNK pathway in response to osmotic stress. Sci. Adv. 11, eadv4636 40668933 10.1126/sciadv.adv4636 PMC12266122

[92] Magana-Avila G. and Castaneda-Bueno M. (2025) WNK kinase activity is modulated by the pseudokinase NRBP1 and the scaffold proteins of the TSC22D family. Curr. Opin. Nephrol. Hypertens. 34, 408–414 40726374 10.1097/MNH.0000000000001097

[93] Magaña-Ávila G., Carbajal-Contreras H., Amnekar R.V., Dite T., Téllez-Sutterlin M., García-Ávila K. et al. (2025) NRBP1 and TSC22D proteins affect distal convoluted tubule physiology through modulation of the WNK pathway. Sci. Adv. 11, eadv2083 40668923 10.1126/sciadv.adv2083 PMC12266109

[94] Mahrour N., Redwine W.B., Florens L., Swanson S.K., Martin-Brown S., Bradford W.D. et al. (2008) Characterization of Cullin-box sequences that direct recruitment of Cul2-Rbx1 and Cul5-Rbx2 modules to Elongin BC-based ubiquitin ligases. J. Biol. Chem. 283, 8005–8013 18187417 10.1074/jbc.M706987200

[95] Zambrowicz B.P., Abuin A., Ramirez-Solis R., Richter L.J., Piggott J., BeltrandelRio H. et al. (2003) Wnk1 kinase deficiency lowers blood pressure in mice: a gene-trap screen to identify potential targets for therapeutic intervention. Proc. Natl. Acad. Sci. U.S.A. 100, 14109–14114 14610273 10.1073/pnas.2336103100 PMC283554

[96] Xie J., Yoon J., Yang S.S., Lin S.H. and Huang C.L. (2013) WNK1 protein kinase regulates embryonic cardiovascular development through the OSR1 signaling cascade. J. Biol. Chem. 288, 8566–8574 23386621 10.1074/jbc.M113.451575 PMC3605675

[97] Park H.W., Kim J.Y., Choi S.K., Lee Y.H., Zeng W., Kim K.H. et al. (2011) Serine-threonine kinase with-no-lysine 4 (WNK4) controls blood pressure via transient receptor potential canonical 3 (TRPC3) in the vasculature. Proc. Natl. Acad. Sci. U.S.A. 108, 10750–10755 21670282 10.1073/pnas.1104271108 PMC3127915

[98] Lalioti M.D., Zhang J., Volkman H.M., Kahle K.T., Hoffmann K.E., Toka H.R. et al. (2006) Wnk4 controls blood pressure and potassium homeostasis via regulation of mass and activity of the distal convoluted tubule. Nat. Genet. 38, 1124–1132 16964266 10.1038/ng1877

[99] Verissimo F. and Jordan P. (2001) WNK kinases, a novel protein kinase subfamily in multi-cellular organisms. Oncogene 20, 5562–5569 11571656 10.1038/sj.onc.1204726

[100] Sun L., Gao M., Yang G.Y., Lu F.T., Liang Z.J., Guo K.M. et al. (2025) ClC-5 knockout mitigates angiotensin II-induced hypertension and endothelial dysfunction. Life Sci. 362, 123342 39740756 10.1016/j.lfs.2024.123342

[101] Garrud T.A.C., Bell B., Mata-Daboin A., Peixoto-Neves D., Collier D.M., Cordero-Morales J.F. et al. (2024) WNK kinase is a vasoactive chloride sensor in endothelial cells. Proc. Natl. Acad. Sci. U.S.A. 121, e2322135121 38568964 10.1073/pnas.2322135121 PMC11009681

[102] Dai J., Sheetz M.P., Wan X. and Morris C.E. (1998) Membrane tension in swelling and shrinking molluscan neurons. J. Neurosci. 18, 6681–6692 9712640 10.1523/JNEUROSCI.18-17-06681.1998 PMC6792972

[103] Roffay C., Molinard G., Kim K., Urbanska M., Andrade V., Barbarasa V. et al. (2021) Passive coupling of membrane tension and cell volume during active response of cells to osmosis. Proc. Natl. Acad. Sci. U.S.A. 118, e2103228118 34785592 10.1073/pnas.2103228118 PMC8617515

[104] Flatman P.W. (2007) Cotransporters, WNKs and hypertension: important leads from the study of monogenetic disorders of blood pressure regulation. Clin. Sci. (Lond.) 112, 203–216 17223794 10.1042/CS20060225

[105] Dowd B.F. and Forbush B. (2003) PASK (proline-alanine-rich STE20-related kinase), a regulatory kinase of the Na-K-Cl cotransporter (NKCC1). J. Biol. Chem. 278, 27347–27353 12740379 10.1074/jbc.M301899200

[106] Richardson C., Rafiqi F.H., Karlsson H.K., Moleleki N., Vandewalle A., Campbell D.G. et al. (2008) Activation of the thiazide-sensitive Na^+^-Cl^-^ cotransporter by the WNK-regulated kinases SPAK and OSR1. J. Cell Sci. 121, 675–684 18270262 10.1242/jcs.025312

[107] Richardson C., Sakamoto K., de los Heros P., Deak M., Campbell D.G., Prescott A.R. et al. (2011) Regulation of the NKCC2 ion cotransporter by SPAK-OSR1-dependent and -independent pathways. J. Cell Sci. 124, 789–800 21321328 10.1242/jcs.077230 PMC3114804

[108] Vitari A.C., Thastrup J., Rafiqi F.H., Deak M., Morrice N.A., Karlsson H.K. et al. (2006) Functional interactions of the SPAK/OSR1 kinases with their upstream activator WNK1 and downstream substrate NKCC1. Biochem. J. 397, 223–231 16669787 10.1042/BJ20060220 PMC1479760

[109] Friedel P., Kahle K.T., Zhang J., Hertz N., Pisella L.I., Buhler E. et al. (2015) WNK1-regulated inhibitory phosphorylation of the KCC2 cotransporter maintains the depolarizing action of GABA in immature neurons. Sci. Signal. 8, ra65 26126716 10.1126/scisignal.aaa0354

[110] Zook E., Pan Y.E., Wipplinger A., Kerschbaum H.H., Clements R.J., Ritter M. et al. (2024) Delayed vacuolation in mammalian cells caused by hypotonicity and ion loss. Sci. Rep. 14, 29354 39592718 10.1038/s41598-024-79815-z PMC11599563

[111] Zhou J., Li Y.S. and Chien S. (2014) Shear stress-initiated signaling and its regulation of endothelial function. Arterioscler. Thromb. Vasc. Biol. 34, 2191–2198 24876354 10.1161/ATVBAHA.114.303422 PMC4169328

[112] Chien S. (2007) Mechanotransduction and endothelial cell homeostasis: the wisdom of the cell. Am. J. Physiol. Heart Circ. Physiol. 292, H1209–H1224 17098825 10.1152/ajpheart.01047.2006

[113] Davies P.F., Civelek M., Fang Y. and Fleming I. (2013) The atherosusceptible endothelium: endothelial phenotypes in complex haemodynamic shear stress regions *in vivo*. Cardiovasc. Res. 99, 315–327 23619421 10.1093/cvr/cvt101 PMC3695748

[114] Roman B.L. and Pekkan K. (2012) Mechanotransduction in embryonic vascular development. Biomech. Model. Mechanobiol. 11, 1149–1168 22744845 10.1007/s10237-012-0412-9 PMC4502581

[115] Dhawan S.S., Avati Nanjundappa R.P., Branch J.R., Taylor W.R., Quyyumi A.A., Jo H. et al. (2010) Shear stress and plaque development. Expert Rev. Cardiovasc. Ther. 8, 545–556 20397828 10.1586/erc.10.28 PMC5467309

[116] Cunningham K.S. and Gotlieb A.I. (2005) The role of shear stress in the pathogenesis of atherosclerosis. Lab. Invest. 85, 9–23 15568038 10.1038/labinvest.3700215

[117] Samady H., Eshtehardi P., McDaniel M.C., Suo J., Dhawan S.S., Maynard C. et al. (2011) Coronary artery wall shear stress is associated with progression and transformation of atherosclerotic plaque and arterial remodeling in patients with coronary artery disease. Circulation 124, 779–788 21788584 10.1161/CIRCULATIONAHA.111.021824

[118] Bowley G., Irving S., Hoefer I., Wilkinson R., Pasterkamp G., Darwish H.M.S. et al. (2024) Zebrafish model for functional screening of flow-responsive genes controlling endothelial cell proliferation. Sci. Rep. 14, 30130 39627337 10.1038/s41598-024-77370-1 PMC11615307

[119] Habib K., Sultan M.T., Jahan I., Rahman M.S., Kundu S.K., Sarker B. et al. (2025) Pharmacological inhibition of SPAK-NKCC1 complex attenuates astrogliosis and restores cerebral blood flow in a mouse model of VCID. Acta Neuropathol. Commun. 13, 216 41137045 10.1186/s40478-025-02137-2 PMC12551359

[120] Lai J.G., Tsai S.M., Tu H.C., Chen W.C., Kou F.J., Lu J.W. et al. (2014) Zebrafish WNK lysine deficient protein kinase 1 (wnk1) affects angiogenesis associated with VEGF signaling. PLoS One 9, e106129 25171174 10.1371/journal.pone.0106129 PMC4149531

[121] Davies P.F. (1995) Flow-mediated endothelial mechanotransduction. Physiol. Rev. 75, 519–560 7624393 10.1152/physrev.1995.75.3.519 PMC3053532

[122] Baeyens N., Nicoli S., Coon B.G., Ross T.D., Van den Dries K., Han J. et al. (2015) Vascular remodeling is governed by a VEGFR3-dependent fluid shear stress set point. eLife 4, e04645 25643397 10.7554/eLife.04645 PMC4337723

[123] Li J., Hou B., Tumova S., Muraki K., Bruns A., Ludlow M.J. et al. (2014) Piezo1 integration of vascular architecture with physiological force. Nature 515, 279–282 25119035 10.1038/nature13701 PMC4230887

[124] Andolfo I., Monaco V., Cozzolino F., Rosato B.E., Marra R., Cerbone V. et al. (2023) Proteome alterations in erythrocytes with PIEZO1 gain-of-function mutations. Blood Adv. 7, 2681–2693 36595486 10.1182/bloodadvances.2022008673 PMC10333744

[125] Leonardi M.V., Di Battista A., Donati L., Sforna L., Morena F., Di Cristina M. et al. (2025) Piezo1 is an essential player in volume regulation of human glioblastoma cells. J. Physiol. 603, 4765–4784 40838979 10.1113/JP289215 PMC12400783

[126] Christensen A.P. and Corey D.P. (2007) TRP channels in mechanosensation: direct or indirect activation? Nat. Rev. Neurosci. 8, 510–521 17585304 10.1038/nrn2149

[127] Solano A.S., Lavanderos B., Metwally E. and Earley S. (2025) Transient receptor potential channels in vascular mechanotransduction. Am. J. Hypertens. 38, 151–160 39579078 10.1093/ajh/hpae134 PMC13368814

[128] Mendoza S.A., Fang J., Gutterman D.D., Wilcox D.A., Bubolz A.H., Li R. et al. (2010) TRPV4-mediated endothelial Ca^2+^ influx and vasodilation in response to shear stress. Am. J. Physiol. Heart Circ. Physiol. 298, H466–H476 19966050 10.1152/ajpheart.00854.2009 PMC2822567

[129] Andrade Y.N., Fernandes J., Vazquez E., Fernandez-Fernandez J.M., Arniges M., Sanchez T.M. et al. (2005) TRPV4 channel is involved in the coupling of fluid viscosity changes to epithelial ciliary activity. J. Cell Biol. 168, 869–874 15753126 10.1083/jcb.200409070 PMC2171792

[130] Ryskamp D.A., Frye A.M., Phuong T.T., Yarishkin O., Jo A.O., Xu Y. et al. (2016) TRPV4 regulates calcium homeostasis, cytoskeletal remodeling, conventional outflow and intraocular pressure in the mammalian eye. Sci. Rep. 6, 30583 27510430 10.1038/srep30583 PMC4980693

[131] Swain S.M. and Liddle R.A. (2021) Piezo1 acts upstream of TRPV4 to induce pathological changes in endothelial cells due to shear stress. J. Biol. Chem. 296, 100171 33298523 10.1074/jbc.RA120.015059 PMC7948745

[132] Fu Y., Subramanya A., Rozansky D. and Cohen D.M. (2006) WNK kinases influence TRPV4 channel function and localization. Am. J. Physiol. Renal. Physiol. 290, F1305–F1314 16403833 10.1152/ajprenal.00391.2005

[133] Tomilin V.N., Pyrshev K., Khayyat N.H., Zaika O. and Pochynyuk O. (2021) With-no-lysine kinase 1 (WNK1) augments TRPV4 function in the aldosterone-sensitive distal nephron. Cells 10, 1482 34204757 10.3390/cells10061482 PMC8231605

[134] Erfinanda L., Zou L., Gutbier B., Kneller L., Weidenfeld S., Michalick L. et al. (2022) Loss of endothelial CFTR drives barrier failure and edema formation in lung infection and can be targeted by CFTR potentiation. Sci. Transl. Med. 14, eabg8577 36475904 10.1126/scitranslmed.abg8577

[135] Chen Y.S., Lu M.J., Huang H.S. and Ma M.C. (2010) Mechanosensitive transient receptor potential vanilloid type 1 channels contribute to vascular remodeling of rat fistula veins. J. Vasc. Surg. 52, 1310–1320 20638226 10.1016/j.jvs.2010.05.095

[136] Castrejon-Tellez V., Del Valle-Mondragon L., Perez-Torres I., Guarner-Lans V., Pastelin-Hernandez G., Ruiz-Ramirez A. et al. (2022) TRPV1 contributes to modulate the nitric oxide pathway and oxidative stress in the isolated and perfused rat heart during ischemia and reperfusion. Molecules 27, 1031 35164296 10.3390/molecules27031031 PMC8839190

[137] Shahidullah M., Mandal A. and Delamere N.A. (2018) Activation of TRPV1 channels leads to stimulation of NKCC1 cotransport in the lens. Am. J. Physiol. Cell Physiol. 315, C793–C802 30207782 10.1152/ajpcell.00252.2018 PMC6336939

[138] Hoover R.S., Tomilin V., Hanson L., Pochynyuk O. and Ko B. (2016) PTH modulation of NCC activity regulates TRPV5 Ca2+ reabsorption. Am. J. Physiol. Renal. Physiol. 310, F144–F151 26608788 10.1152/ajprenal.00323.2015 PMC4719045

[139] An S.W., Cha S.K., Yoon J., Chang S., Ross E.M. and Huang C.L. (2011) WNK1 promotes PIP_2_ synthesis to coordinate growth factor and GPCR-Gq signaling. Curr. Biol. 21, 1979–1987 22119528 10.1016/j.cub.2011.11.002 PMC3237931

[140] Kim J.H., Hwang K.H., Eom M., Kim M., Park E.Y., Jeong Y. et al. (2019) WNK1 promotes renal tumor progression by activating TRPC6-NFAT pathway. FASEB J. 33, 8588–8599 31022353 10.1096/fj.201802019RR

[141] Miyamoto T., Mochizuki T., Nakagomi H., Kira S., Watanabe M., Takayama Y. et al. (2014) Functional role for Piezo1 in stretch-evoked Ca^2+^ influx and ATP release in urothelial cell cultures. J. Biol. Chem. 289, 16565–16575 24759099 10.1074/jbc.M113.528638 PMC4047422

[142] Servin-Vences M.R., Moroni M., Lewin G.R. and Poole K. (2017) Direct measurement of TRPV4 and PIEZO1 activity reveals multiple mechanotransduction pathways in chondrocytes. eLife 6, e21074 28135189 10.7554/eLife.21074 PMC5279942

[143] Rong S., Zhang L., Wang J. and Dong H. (2024) Regulatory role of Piezo1 channel in endothelium-dependent hyperpolarization-mediated vasorelaxation of small resistance vessels and its anti-inflammatory action. Life Sci. 336, 122326 38056769 10.1016/j.lfs.2023.122326

[144] Endesh N., Chuntharpursat-Bon E., Revill C., Yuldasheva N.Y., Futers T.S., Parsonage G. et al. (2023) Independent endothelial functions of PIEZO1 and TRPV4 in hepatic portal vein and predominance of PIEZO1 in mechanical and osmotic stress. Liver Int. 43, 2026–2038 37349903 10.1111/liv.15646 PMC10946873

[145] Swain S.M., Romac J.M., Shahid R.A., Pandol S.J., Liedtke W., Vigna S.R. et al. (2020) TRPV4 channel opening mediates pressure-induced pancreatitis initiated by Piezo1 activation. J. Clin. Invest. 130, 2527–2541 31999644 10.1172/JCI134111 PMC7190979

[146] Guntur D., Jeremic D., Csaki R., Myronenko O., Biasin V., Kolb D. et al. (2025) BK channels are indispensable for endothelial function in small pulmonary arteries. Cell Commun. Signal. 23, 448 41121159 10.1186/s12964-025-02436-0 PMC12542031

[147] Allerkamp H.H., Bondarenko A.I., Tawfik I., Kamali-Simsek N., Horvat Mercnik M., Madreiter-Sokolowski C.T. et al. (2025) *In vitro* examination of Piezo1-TRPV4 dynamics: implications for placental endothelial function in normal and preeclamptic pregnancies. Am. J. Physiol. Cell Physiol. 328, C227–C244 39652778 10.1152/ajpcell.00794.2024 PMC7617947

[148] Yundung Y., Pelisek J., Maccio U., Varga Z., Ech-Cherif H., Markkanen E. et al. (2025) Identification of putative baroreceptors in human aortic arch by histological and omics analyses. Hypertens. Res. 48, 2083–2094 40335722 10.1038/s41440-025-02217-9 PMC12229889

[149] Sonkusare S.K., Dalsgaard T., Bonev A.D. and Nelson M.T. (2016) Inward rectifier potassium (Kir2.1) channels as end-stage boosters of endothelium-dependent vasodilators. J. Physiol. 594, 3271–3285 26840527 10.1113/JP271652 PMC4908010

[150] Hoger J.H., Ilyin V.I., Forsyth S. and Hoger A. (2002) Shear stress regulates the endothelial Kir2.1 ion channel. Proc. Natl. Acad. Sci. U.S.A. 99, 7780–7785 12032360 10.1073/pnas.102184999 PMC124350

[151] He Y., Xiao J., Yang Y., Zhou Q., Zhang Z., Pan Q. et al. (2006) Stretch-induced alterations of human Kir2.1 channel currents. Biochem. Biophys. Res. Commun. 351, 462–467 17067550 10.1016/j.bbrc.2006.10.049

[152] Jaykumar A.B., Plumber S., Barry D.M., Binns D., Wichaidit C., Grzemska M. et al. (2022) WNK1 collaborates with TGF-β in endothelial cell junction turnover and angiogenesis. Proc. Natl. Acad. Sci. U.S.A. 119, e2203743119 35867836 10.1073/pnas.2203743119 PMC9335306

[153] Ishizawa K., Kaseda K., Fujii W., Tomomitsu Y., Hirohama D., Yamazaki O. et al. (2026) Piezo1 dictates K(+) homeostasis through coordinated regulation of the ubiquitin ligase Kelch-like 3 in RBCs and the kidney. Proc. Natl. Acad. Sci. U.S.A. 123, e2513222123 41533447 10.1073/pnas.2513222123 PMC12818455

[154] Bergaya S., Faure S., Baudrie V., Rio M., Escoubet B., Bonnin P. et al. (2011) WNK1 regulates vasoconstriction and blood pressure response to alpha 1-adrenergic stimulation in mice. Hypertension 58, 439–445 21768522 10.1161/HYPERTENSIONAHA.111.172429

[155] Susa K., Kita S., Iwamoto T., Yang S.S., Lin S.H., Ohta A. et al. (2012) Effect of heterozygous deletion of WNK1 on the WNK-OSR1/ SPAK-NCC/NKCC1/NKCC2 signal cascade in the kidney and blood vessels. Clin. Exp. Nephrol. 16, 530–538 22294159 10.1007/s10157-012-0590-x

[156] Quelquejay H., Al-Rifai R., Silvestro M., Vandestienne M., Ferreira I., Mirault T. et al. (2024) L-Wnk1 deletion in smooth muscle cells causes aortitis and inflammatory shift. Circ. Res. 135, 488–502 38979610 10.1161/CIRCRESAHA.124.324366

[157] Zeniya M., Sohara E., Kita S., Iwamoto T., Susa K., Mori T. et al. (2013) Dietary salt intake regulates WNK3-SPAK-NKCC1 phosphorylation cascade in mouse aorta through angiotensin II. Hypertension 62, 872–878 24019400 10.1161/HYPERTENSIONAHA.113.01543

[158] Lu F.T., Huang C.C., Lai W.Y., Yang G.Y., Liang Z.J., Zhang Z.Y. et al. (2024) Vascular smooth muscle-specific LRRC8A knockout ameliorates angiotensin II-induced cerebrovascular remodeling by inhibiting the WNK1/FOXO3a/MMP signaling pathway. Acta Pharmacol. Sin. 45, 1848–1860 38719954 10.1038/s41401-024-01280-1 PMC11335743

[159] Nishida H., Sohara E., Nomura N., Chiga M., Alessi D.R., Rai T. et al. (2012) Phosphatidylinositol 3-kinase/Akt signaling pathway activates the WNK-OSR1/SPAK-NCC phosphorylation cascade in hyperinsulinemic db/db mice. Hypertension 60, 981–990 22949526 10.1161/HYPERTENSIONAHA.112.201509 PMC3743028

[160] Komers R., Rogers S., Oyama T.T., Xu B., Yang C.L., McCormick J. et al. (2012) Enhanced phosphorylation of Na(+)-Cl- co-transporter in experimental metabolic syndrome: role of insulin. Clin. Sci. (Lond.) 123, 635–647 22651238 10.1042/CS20120003 PMC3943429

[161] Mendes A.I., Matos P., Moniz S. and Jordan P. (2010) Protein kinase WNK1 promotes cell surface expression of glucose transporter GLUT1 by regulating a Tre-2/USP6-BUB2-Cdc16 domain family member 4 (TBC1D4)-Rab8A complex. J. Biol. Chem. 285, 39117–39126 20937822 10.1074/jbc.M110.159418 PMC2998075

[162] Jaykumar A.B., Binns D., Taylor C.A. 4th, Anselmo A., Kankanamalage S.G., Birnbaum S.G. et al. (2025) WNKs regulate mouse behavior and alter central nervous system glucose uptake and insulin signaling. eLife 13, RP100097 10.7554/eLife.100097

[163] Kim J.H., Kim H., Hwang K.H., Chang J.S., Park K.S., Cha S.K. et al. (2018) WNK1 kinase is essential for insulin-stimulated GLUT4 trafficking in skeletal muscle. FEBS Open Bio 8, 1866–1874 30410865 10.1002/2211-5463.12528 PMC6212645

[164] Ding L., Zhang L., Biswas S., Schugar R.C., Brown J.M., Byzova T. et al. (2017) Akt3 inhibits adipogenesis and protects from diet-induced obesity via WNK1/SGK1 signaling. JCI Insight 2, e95687 29202451 10.1172/jci.insight.95687 PMC5752373

[165] Robbins D.J., Zhen E., Owaki H., Vanderbilt C.A., Ebert D., Geppert T.D. et al. (1993) Regulation and properties of extracellular signal-regulated protein kinases 1 and 2 *in vitro*. J. Biol. Chem. 268, 5097–5106 8444886 10.1016/S0021-9258(18)53507-9

[166] Takahashi D., Mori T., Sohara E., Tanaka M., Chiga M., Inoue Y. et al. (2017) WNK4 is an adipogenic factor and its deletion reduces diet-induced obesity in mice. eBioMedicine 18, 118–127 28314693 10.1016/j.ebiom.2017.03.011 PMC5405161

[167] Rodan A.R. (2018) WNK-SPAK/OSR1 signaling: lessons learned from an insect renal epithelium. Am. J. Physiol. Renal. Physiol. 315, F903–F907 29923766 10.1152/ajprenal.00176.2018 PMC6230732

[168] Guo Q., Zhang Y., Jiang G.R. and Zhang C. (2021) Decreased KLHL3 expression is involved in the activation of WNK-OSR1/SPAK-NCC cascade in type 1 diabetic mice. Pflugers Arch. 473, 185–196 33432425 10.1007/s00424-020-02509-8

[169] Zhang Y., Guo Q., Jiang G. and Zhang C. (2021) Dysfunction of Cullin 3 RING E3 ubiquitin ligase causes vasoconstriction and increased sodium reabsorption in diabetes. Arch. Biochem. Biophys. 710, 109000 34343486 10.1016/j.abb.2021.109000

[170] Jin X., Xie J., Yeh C.W., Li Y.J., Lien C.C. and Huang C.L. (2025) Central osmolality sensing for arginine vasopressin release is mediated by WNK1-OSR1/SPAK-Kv3.1 cascade. FASEB J. 39, e71213 41246868 10.1096/fj.202502072RR PMC12621171

[171] Jin X., Xie J., Yeh C.W., Chen J.C., Cheng C.J., Lien C.C. et al. (2023) WNK1 promotes water homeostasis by acting as a central osmolality sensor for arginine vasopressin release. J. Clin. Invest. 133, e164222 37071482 10.1172/JCI164222 PMC10231991

[172] Geng Y., Hoke A. and Delpire E. (2009) The Ste20 kinases Ste20-related proline-alanine-rich kinase and oxidative-stress response 1 regulate NKCC1 function in sensory neurons. J. Biol. Chem. 284, 14020–14028 19307180 10.1074/jbc.M900142200 PMC2682850

[173] Josiah S.S., Meor Azlan N.F. and Zhang J. (2021) Targeting the WNK-SPAK/OSR1 pathway and cation-chloride cotransporters for the therapy of stroke. Int. J. Mol. Sci. 22, 1232 33513812 10.3390/ijms22031232 PMC7865768

[174] Eick A.K., Ogueta M., Buhl E., Hodge J.J.L. and Stanewsky R. (2022) The opposing chloride cotransporters KCC and NKCC control locomotor activity in constant light and during long days. Curr. Biol. 32, 1420.e1424–1428.e1424 35303416 10.1016/j.cub.2022.01.056

[175] McMoneagle E., Zhou J., Zhang S., Huang W., Josiah S.S., Ding K. et al. (2024) Neuronal K^+^-Cl^-^ cotransporter KCC2 as a promising drug target for epilepsy treatment. Acta Pharmacol. Sin. 45, 1–22 37704745 10.1038/s41401-023-01149-9 PMC10770335

[176] Alessi D.R., Zhang J., Khanna A., Hochdorfer T., Shang Y. and Kahle K.T. (2014) The WNK-SPAK/OSR1 pathway: master regulator of cation-chloride cotransporters. Sci. Signal. 7, re3 25028718 10.1126/scisignal.2005365

[177] Shekarabi M., Zhang J., Khanna A.R., Ellison D.H., Delpire E. and Kahle K.T. (2017) WNK kinase signaling in ion homeostasis and human disease. Cell Metab. 25, 285–299 28178566 10.1016/j.cmet.2017.01.007

[178] Furukawa T. and Fukuda A. (2023) Maternal taurine as a modulator of Cl^-^ homeostasis as well as of glycine/GABA(A) receptors for neocortical development. Front. Cell. Neurosci. 17, 1221441 37601283 10.3389/fncel.2023.1221441 PMC10435090

[179] Arion D. and Lewis D.A. (2011) Altered expression of regulators of the cortical chloride transporters NKCC1 and KCC2 in schizophrenia. Arch. Gen. Psychiatry 68, 21–31 20819979 10.1001/archgenpsychiatry.2010.114 PMC3015012

[180] Rinehart J., Vazquez N., Kahle K.T., Hodson C.A., Ring A.M., Gulcicek E.E. et al. (2011) WNK2 kinase is a novel regulator of essential neuronal cation-chloride cotransporters. J. Biol. Chem. 286, 30171–30180 21733846 10.1074/jbc.M111.222893 PMC3191056

[181] Yang H.J., Kim M.J., Kim S.S. and Cho Y.W. (2021) Melatonin modulates nitric oxide-regulated WNK-SPAK/OSR1-NKCC1 signaling in dorsal raphe nucleus of rats. Korean J. Physiol. Pharmacol. 25, 449–457 34448462 10.4196/kjpp.2021.25.5.449 PMC8405441

[182] Amakhin D.V., Ergina J.L., Chizhov A.V. and Zaitsev A.V. (2016) Synaptic conductances during interictal discharges in pyramidal neurons of rat entorhinal cortex. Front. Cell. Neurosci. 10, 233 27790093 10.3389/fncel.2016.00233 PMC5061778

[183] Lee K.L., Abiraman K., Lucaj C., Ollerhead T.A., Brandon N.J., Deeb T.Z. et al. (2022) Inhibiting with-no-lysine kinases enhances K+/Cl- cotransporter 2 activity and limits status epilepticus. Brain 145, 950–963 34528073 10.1093/brain/awab343 PMC9050525

[184] Andrews J.L. and Fernandez-Enright F. (2015) Genetic variants in Nogo receptor signaling pathways may be associated with early life adversity in schizophrenia susceptibility. BBA Clin. 3, 36–43 26673096 10.1016/j.bbacli.2014.11.008 PMC4661513

[185] Yang C.L., Angell J., Mitchell R. and Ellison D.H. (2003) WNK kinases regulate thiazide-sensitive Na-Cl cotransport. J. Clin. Invest. 111, 1039–1045 12671053 10.1172/JCI17443 PMC152590

[186] Elvira B., Singh Y., Warsi J., Munoz C. and Lang F. (2016) OSR1 and SPAK sensitivity of large-conductance Ca^2+^ activated K^+^ channel. Cell. Physiol. Biochem. 38, 1652–1662 27119824 10.1159/000443105

[187] Abousaab A., Warsi J., Elvira B., Alesutan I., Hoseinzadeh Z. and Lang F. (2015) Down-regulation of excitatory amino acid transporters EAAT1 and EAAT2 by the kinases SPAK and OSR1. J. Membr. Biol. 248, 1107–1119 26233565 10.1007/s00232-015-9826-5

[188] Shimizu M. and Shibuya H. (2022) WNK1/HSN2 mediates neurite outgrowth and differentiation via a OSR1/GSK3β-LHX8 pathway. Sci. Rep. 12, 15858 36151370 10.1038/s41598-022-20271-y PMC9508073

[189] Begum G., Yuan H., Kahle K.T., Li L., Wang S., Shi Y. et al. (2015) Inhibition of WNK3 kinase signaling reduces brain damage and accelerates neurological recovery after stroke. Stroke 46, 1956–1965 26069258 10.1161/STROKEAHA.115.008939 PMC4643659

[190] Huang H., Song S., Banerjee S., Jiang T., Zhang J., Kahle K.T. et al. (2019) The WNK-SPAK/OSR1 kinases and the cation-chloride cotransporters as therapeutic targets for neurological diseases. Aging Dis. 10, 626–636 31165006 10.14336/AD.2018.0928 PMC6538211

[191] Zhao H., Nepomuceno R., Gao X., Foley L.M., Wang S., Begum G. et al. (2017) Deletion of the WNK3-SPAK kinase complex in mice improves radiographic and clinical outcomes in malignant cerebral edema after ischemic stroke. J. Cereb. Blood Flow Metab. 37, 550–563 26861815 10.1177/0271678X16631561 PMC5381450

[192] Bhuiyan M.I.H., Young C.B., Jahan I., Hasan M.N., Fischer S., Meor Azlan N.F. et al. (2022) NF-κB signaling-mediated activation of WNK-SPAK-NKCC1 cascade in worsened stroke outcomes of Ang II-hypertensive mice. Stroke 53, 1720–1734 35272484 10.1161/STROKEAHA.121.038351 PMC9038703

[193] Zhao P., Feng H., Zhou X., Zhou J., Hu F., Hu T. et al. (2025) WNK1 alleviates chloride efflux-induced NLRP3 inflammasome activation and subsequent neuroinflammation in early brain injury following subarachnoid hemorrhage. Neurosci. Bull. 41, 1570–1588 40419746 10.1007/s12264-025-01414-3 PMC12433386

[194] Mayes-Hopfinger L., Enache A., Xie J., Huang C.L., Kochl R., Tybulewicz V.L.J. et al. (2021) Chloride sensing by WNK1 regulates NLRP3 inflammasome activation and pyroptosis. Nat. Commun. 12, 4546 34315884 10.1038/s41467-021-24784-4 PMC8316491

[195] Tian Y., Wang Y., Zhao Y., Liu C., Zhang X., Zhang Y. et al. (2025) LRRC8A in endothelial cells contributes to the aberrant blood-brain barrier integrity in ischaemic stroke. Stroke Vasc. Neurol. 10, 625–636 40096965 10.1136/svn-2024-003675 PMC12573346

[196] Bhuiyan M.I.H., Song S., Yuan H., Begum G., Kofler J., Kahle K.T. et al. (2017) WNK-Cab39-NKCC1 signaling increases the susceptibility to ischemic brain damage in hypertensive rats. J. Cereb. Blood Flow Metab. 37, 2780–2794 27798271 10.1177/0271678X16675368 PMC5536788

[197] Bercier V., Brustein E., Liao M., Dion P.A., Lafreniere R.G., Rouleau G.A. et al. (2013) WNK1/HSN2 mutation in human peripheral neuropathy deregulates KCC2 expression and posterior lateral line development in zebrafish (*Danio rerio*). PLoS Genet. 9, e1003124 23300475 10.1371/journal.pgen.1003124 PMC3536653

[198] Lafreniere R.G., MacDonald M.L., Dube M.P., MacFarlane J., O'Driscoll M., Brais B. et al. (2004) Identification of a novel gene (HSN2) causing hereditary sensory and autonomic neuropathy type II through the Study of Canadian Genetic Isolates. Am. J. Hum. Genet. 74, 1064–1073 15060842 10.1086/420795 PMC1181970

[199] Coen K., Pareyson D., Auer-Grumbach M., Buyse G., Goemans N., Claeys K.G. et al. (2006) Novel mutations in the HSN2 gene causing hereditary sensory and autonomic neuropathy type II. Neurology 66, 748–751 16534117 10.1212/01.wnl.0000201191.57519.47

[200] Kahle K.T. and Delpire E. (2016) Kinase-KCC2 coupling: Cl- rheostasis, disease susceptibility, therapeutic target. J. Neurophysiol. 115, 8–18 26510764 10.1152/jn.00865.2015 PMC4760510

[201] Jung J.U., Jaykumar A.B. and Cobb M.H. (2022) WNK1 in malignant behaviors: a potential target for cancer? Front. Cell Dev. Biol. 10, 935318 35813203 10.3389/fcell.2022.935318 PMC9257110

[202] Chen L., Jenjaroenpun P., Pillai A.M., Ivshina A.V., Ow G.S., Efthimios M. et al. (2017) Transposon insertional mutagenesis in mice identifies human breast cancer susceptibility genes and signatures for stratification. Proc. Natl. Acad. Sci. U.S.A. 114, E2215–E2224 28251929 10.1073/pnas.1701512114 PMC5358385

[203] Zhou B., Min B., Liu W., Li Y., Zhu F., Huang J. et al. (2024) Construction of a five-gene-based prognostic model for relapsed/refractory acute lymphoblastic leukemia. Hematology 29, 2412952 39453390 10.1080/16078454.2024.2412952

[204] Hwang I.Y., Kim J.S., Harrison K.A., Park C., Shi C.S. and Kehrl J.H. (2024) Chemokine-mediated F-actin dynamics, polarity, and migration in B lymphocytes depend on WNK1 signaling. Sci. Signal. 17, eade1119 39190707 10.1126/scisignal.ade1119 PMC11542683

[205] Hong C., Moorefield K.S., Jun P., Aldape K.D., Kharbanda S., Phillips H.S. et al. (2007) Epigenome scans and cancer genome sequencing converge on WNK2, a kinase-independent suppressor of cell growth. Proc. Natl. Acad. Sci. U.S.A. 104, 10974–10979 17578925 10.1073/pnas.0700683104 PMC1904113

[206] Costa V., Esposito R., Ziviello C., Sepe R., Bim L.V., Cacciola N.A. et al. (2015) New somatic mutations and WNK1-B4GALNT3 gene fusion in papillary thyroid carcinoma. Oncotarget 6, 11242–11251 25803323 10.18632/oncotarget.3593 PMC4484453

[207] Liu Z.L., Chen H.H., Zheng L.L., Sun L.P. and Shi L. (2023) Angiogenic signaling pathways and anti-angiogenic therapy for cancer. Signal Transduct. Target Ther. 8, 198 37169756 10.1038/s41392-023-01460-1 PMC10175505

[208] Weis S.M. and Cheresh D.A. (2011) Tumor angiogenesis: molecular pathways and therapeutic targets. Nat. Med. 17, 1359–1370 22064426 10.1038/nm.2537

[209] Hou C.Y., Ma C.Y., Lin Y.J., Huang C.L., Wang H.D. and Yuh C.H. (2022) WNK1-OSR1 signaling regulates angiogenesis-mediated metastasis towards developing a combinatorial anti-cancer strategy. Int. J. Mol. Sci. 23, 12100 36292952 10.3390/ijms232012100 PMC9602556

[210] Sie Z.L., Li R.Y., Sampurna B.P., Hsu P.J., Liu S.C., Wang H.D. et al. (2020) WNK1 kinase stimulates angiogenesis to promote tumor growth and metastasis. Cancers (Basel) 12, 575 32131390 10.3390/cancers12030575 PMC7139507

[211] Serra S.A., Stojakovic P., Amat R., Rubio-Moscardo F., Latorre P., Seisenbacher G. et al. (2021) LRRC8A-containing chloride channel is crucial for cell volume recovery and survival under hypertonic conditions. Proc. Natl. Acad. Sci. U.S.A. 118, e2025013118 34083438 10.1073/pnas.2025013118 PMC8201826

[212] Fidaleo A.M., Bach M.D., Orbeta S., Abdullaev I.F., Martino N., Adam A.P. et al. (2026) LRRC8A-containing anion channels promote glioblastoma proliferation via a WNK1/mTORC2-dependent mechanism. J. Physiol. 604, 1995–2026 41636565 10.1113/JP289036 PMC12961766

[213] Duan S., Agger K., Messling J.E., Nishimura K., Han X., Peña-Rømer I. et al. (2025) WNK1 signalling regulates amino acid transport and mTORC1 activity to sustain acute myeloid leukaemia growth. Nat. Commun. 16, 4920 40425534 10.1038/s41467-025-59969-8 PMC12116911

[214] Li X., Li Z., Zhu Y., Li Z., Yao L., Zhang L. et al. (2020) miR-524-5p inhibits angiogenesis through targeting WNK1 in colon cancer cells. Am. J. Physiol. Gastrointest. Liver Physiol. 318, G827–G839 32174132 10.1152/ajpgi.00369.2019

[215] Dong H., Jian P., Yu M. and Wang L. (2020) Silencing of long noncoding RNA LEF1-AS1 prevents the progression of hepatocellular carcinoma via the crosstalk with microRNA-136-5p/WNK1. J. Cell. Physiol. 235, 6548–6562 32068261 10.1002/jcp.29503

[216] Mao Y., Ding Z., Jiang M., Yuan B., Zhang Y. and Zhang X. (2022) Circ_0091579 exerts an oncogenic role in hepatocellular carcinoma via mediating miR-136-5p/TRIM27. Biomed. J. 45, 883–895 34974169 10.1016/j.bj.2021.12.009 PMC9795369

[217] Jung J.U. and Cobb M.H. (2023) WNK1 controls endosomal trafficking through TRIM27-dependent regulation of actin assembly. Proc. Natl. Acad. Sci. U.S.A. 120, e2300310120 37307465 10.1073/pnas.2300310120 PMC10288585

[218] Shyamasundar S., Lim J.P. and Bay B.H. (2016) miR-93 inhibits the invasive potential of triple-negative breast cancer cells *in vitro* via protein kinase WNK1. Int. J. Oncol. 49, 2629–2636 27840899 10.3892/ijo.2016.3761

[219] Fdel A.M., Waters L., Sharma I., Jones S., Gee J., Atack J.R. et al. (2025) Oxidative stress-responsive 1 kinase catalytic activity promotes triple negative breast cancer oncogenic potential. ACS Pharmacol. Transl. Sci. 8, 726–735 40109757 10.1021/acsptsci.4c00603 PMC11915029

[220] Jaykumar A.B., Jung J.U., Parida P.K., Dang T.T., Wichaidit C., Kannangara A.R. et al. (2021) WNK1 enhances migration and invasion in breast cancer models. Mol. Cancer Ther. 20, 1800–1808 34253593 10.1158/1535-7163.MCT-21-0174 PMC9013269

[221] Singh S., Parthasarathi K.T.S., Bhat M.Y., Gopal C., Sharma J. and Pandey A. (2024) Profiling kinase activities for precision oncology in diffuse gastric cancer. OMICS 28, 76–89 38271566 10.1089/omi.2023.0173

[222] Jiang Y., Xiang D., Wen X., He M., Qin Y., Yao X. et al. (2024) ZYX promotes invasion and metastasis of gastric cancer cells via WNK1/SNAI1axis. Genes Dis. 11, 564–567 37692528 10.1016/j.gendis.2023.03.018 PMC10491912

[223] Fang S., Jin G., Yan M., Song Y., Zhao S., Zhang C. et al. (2026) PHGDH phosphorylation mediated by WNK1 serves as a dual marker of metabolic vulnerability and responsiveness to oxaliplatin treatment. Proc. Natl. Acad. Sci. U.S.A. 123, e2525213123 41880577 10.1073/pnas.2525213123 PMC13037954

[224] Li L., Xie D., Yu S., Ma M., Fan K., Chen J. et al. (2024) WNK1 interaction with KEAP1 promotes NRF2 stabilization to enhance the oxidative stress response in hepatocellular carcinoma. Cancer Res. 84, 2776–2791 38885324 10.1158/0008-5472.CAN-23-1167

[225] Tu J. and Zhang L. (2025) LARP4B inhibits ferroptosis and accelerates the progression of pancreatic cancer by activating WNK1-induced NRF2/GCH1/BH4 pathway. Integr. Biol. (Camb) 17, zyaf019 41014504 10.1093/intbio/zyaf019

[226] Hou W., Gad S.A., Ding X., Dhanarajan A. and Qiu W. (2024) Focal adhesion kinase confers lenvatinib resistance in hepatocellular carcinoma via the regulation of lysine-deficient kinase 1. Mol. Carcinog. 63, 173–189 37787401 10.1002/mc.23644 PMC10842616

[227] Xu Z., Wu Y., Yang M., Wei H. and Pu J. (2023) CBX2-mediated suppression of SIAH2 triggers WNK1 accumulations to promote glycolysis in hepatocellular carcinoma. Exp. Cell. Res. 426, 113513 36780970 10.1016/j.yexcr.2023.113513

[228] Ma Y., Zong H., Pan P., Shang H. and Yang X. (2024) The CREB1/WNK1 axis promotes the tumorigenesis of ovarian cancer via regulating HIF-1. Exp. Cell. Res. 438, 114006 38599542 10.1016/j.yexcr.2024.114006

[229] Sanchez-Fdez A., Matilla-Almazan S., Montero J.C., Del Carmen S., Abad M., Garcia-Alonso S. et al. (2023) The WNK1-ERK5 route plays a pathophysiological role in ovarian cancer and limits therapeutic efficacy of trametinib. Clin. Transl. Med. 13, e1217 37029785 10.1002/ctm2.1217 PMC10082568

[230] Ye T., Mishra A.K., Banday S., Li R., Hu K., Coleman M.M. et al. (2024) Identification of WNK1 as a therapeutic target to suppress IgH/MYC expression in multiple myeloma. Cell Rep. 43, 114211 38722741 10.1016/j.celrep.2024.114211

[231] Jung Y.Y., Mohan C.D., Rangappa S., Um J.Y., Chinnathambi A., Alharbi S.A. et al. (2023) Brucein D imparts a growth inhibitory effect in multiple myeloma cells by abrogating the Akt-driven signaling pathway. IUBMB Life 75, 149–160 36262053 10.1002/iub.2684

[232] Mulkearns-Hubert E.E., Hajdari N., Hong E.S., Jacobs A.P., Gaboriau A., Giltner S. et al. (2025) Connexin 43 drives glioblastoma cancer stem cell phenotypes through a WNK lysine-deficient protein kinase 1-c-MYC signaling axis. Cell Rep. 44, 116303 40946315 10.1016/j.celrep.2025.116303 PMC12443334

[233] Zhang H., Wang Z., Qiao X., Peng N., Wu J., Chen Y. et al. (2024) Unveiling the therapeutic potential of IHMT-337 in glioma treatment: targeting the EZH2-SLC12A5 axis. Mol. Med. 30, 91 38886655 10.1186/s10020-024-00857-0 PMC11184773

[234] Sun Q., Lei X., Meng X., Zha C., Yan L. and Zhang W. (2024) Bioinformatics analysis identifies WNK1 gene as a potential biomarker for cholangiocarcinoma diagnosis and immune infiltration. J. Genet. Eng. Biotechnol. 22, 100426 39674642 10.1016/j.jgeb.2024.100426 PMC11465147

[235] Jiang H., Cheng X., Liang Y., Wang Y., Li Y. and Li Y. (2023) Aberrant expression of WNK lysine deficient protein kinase 1 is associated with poor prognosis of colon adenocarcinoma. Ir. J. Med. Sci. 192, 57–64 35138567 10.1007/s11845-021-02916-5

[236] Jin S., Liu T., Wang W., Li T., Liu Z. and Zhang M. (2023) Lymphocyte migration regulation related proteins in urine exosomes may serve as a potential biomarker for lung cancer diagnosis. BMC Cancer 23, 1125 37980468 10.1186/s12885-023-11567-x PMC10656923

[237] Zhang D., Li J., Lu T., Zhao F., Guo P., Li Z. et al. (2024) Illuminating shared genetic associations between oesophageal carcinoma and pulmonary carcinoma risk. J. Cancer. 15, 2412–2423 38495498 10.7150/jca.92899 PMC10937272

[238] Tu S.W., Bugde A., Luby-Phelps K. and Cobb M.H. (2011) WNK1 is required for mitosis and abscission. Proc. Natl. Acad. Sci. U.S.A. 108, 1385–1390 21220314 10.1073/pnas.1018567108 PMC3029763

[239] Steinlein S., Essmann F., Ghilardi A.F., Horn H., Schuler J., Hausser A. et al. (2023) Indolyl-chalcone derivatives trigger apoptosis in cisplatin-resistant mesothelioma cells through aberrant tubulin polymerization and deregulation of microtubule-associated proteins. Front. Oncol. 13, 1190988 37305581 10.3389/fonc.2023.1190988 PMC10248254

[240] Monfort-Vengut A., Sanz-Gómez N., Ballesteros-Sánchez S., Ortigosa B., Cambón A., Ramos M. et al. (2025) Osmotic stress influences microtubule drug response via WNK1 kinase signaling. Drug Resist. Updat. 79, 101203 39855050 10.1016/j.drup.2025.101203

