# Supplementary material for: No lysine? No problem: the surprising roles of WNK kinases in physiology and disease
Source: Biochem J. 2026 Oct 19;483(11):2139–61. doi: 10.1042/BCJ20250110 (PMC13686953; doi:10.1042/BCJ20250110)
Supplement: Supplementary file 1 [file BCJ-2025-0110C_supp.pdf]

Supplemental Table 1. SPAK and OSR1 kinase inhibitors. Summary of information about the properties of known SPAK and OSR1 kinase inhibitors.

Inhibitor	Target / mechanism	Inhibitory activity	Specificity /off-target effects	Ref.
STOCK1S-50699	SPAK CCT domain interaction blocker (likely acts on OSR1 similarly)	Binding inhibition (IC_{50}) between SPAK CCT and… WNK1 peptide: 31 μM WNK4 peptide: 37 μM	---	[91]
STOCK2S-26016	SPAK CCT domain interaction blocker (likely acts on OSR1 similarly)	Binding inhibition (IC_{50}) between SPAK CCT and… WNK1 peptide: 34 μM WNK4 peptide: 16 μM	---	[91]
STOCK1S-14279	non-ATP competitive SPAK inhibitor (likely acts on OSR1 similarly; reported to act as CCT domain interaction blocker in separate publication [94]	IC_{50} (NKCC2 1-174) SPAK: 260 nM	RapidKinaseTM48 specificity panel:<br><br>Inhibition >60% for 1 of 48 kinases tested at 10 μM	[90]
Closantel	non-ATP competitive SPAK inhibitor (likely acts on OSR1 similarly; reported to act as CCT domain interaction blocker in separate publication [94]	IC_{50} (NKCC2 1-174) SPAK: 770 nM	RapidKinaseTM48 specificity panel:<br><br>Inhibition >60% for 2 of 48 kinases tested at 10 μM	[90]
Rafoxanide	SPAK/OSR1 CCT domain interaction blocker	IC_{50} (CATCHtide) OSR1: 8.2 μM<br><br>IC_{50} (CATCHtide) SPAK: 13 μM	---	[89]
ZT-1a	SPAK CCT domain interaction blocker (likely OSR1)	Binding inhibition (IC_{50}) between SPAK CCT and WNK4 peptide: 326 μM<br><br>IC_{50} (CATCHtide) SPAK: 41 μM	International Centre for Kinase Profiling (MRC) kinase panel: 6 of 140 kinases had >50% inhibition at 10 μM	[92]
